# Salvage Chemotherapy with Cisplatin, Ifosfamide, and Paclitaxel in Aggressive Variant of Metastatic Castration-Resistant Prostate Cancer

**DOI:** 10.3390/ijms232314948

**Published:** 2022-11-29

**Authors:** Gunhild von Amsberg, Mirjam Zilles, Wael Mansour, Philipp Gild, Winfried Alsdorf, Moritz Kaune, Lukas Böckelmann, Jessica Hauschild, Christoph Krisp, Tina Rohlfing, Ceren Saygi, Malik Alawi, Alexandra Zielinski, Claudia Langebrake, Su Jung Oh-Hohenhorst, Sven Perner, Derya Tilki, Hartmut Schlüter, Markus Graefen, Sergey A. Dyshlovoy, Carsten Bokemeyer

**Affiliations:** 1 Department of Oncology, University Cancer Center Hamburg, University Medical Center Hamburg-Eppendorf, 20246 Hamburg, Germany; 2 Martini-Klinik Prostate Cancer Center, University Medical Center Hamburg-Eppendorf, 20246 Hamburg, Germany; 3 Department of Radiotherapy and Radiooncology, University Medical Center Hamburg-Eppendorf, 20246 Hamburg, Germany; 4 Mildred Scheel Cancer Career Center HaTriCS4, University Medical Center Hamburg-Eppendorf, 20246 Hamburg, Germany; 5 Department of Urology, University Medical Center Hamburg-Eppendorf, 20246 Hamburg, Germany; 6 Institute of Clinical Chemistry and Laboratory Medicine, Section Mass Spectrometry and Proteomics, University Medical Center Hamburg-Eppendorf, 20246 Hamburg, Germany; 7 Bioinformatics Core, University Medical Center Hamburg-Eppendorf, 20246 Hamburg, Germany; 8 Pharmacy, University Medical Center Hamburg-Eppendorf, 20246 Hamburg, Germany; 9 Department of Urology, Centre Hospitalier de l’Université de Montreal (CHUM)/Centre de recherche du CHUM, Montreal, QC 3840, Canada; 10 Institute of Pathology, University of Lübeck and University Hospital Schleswig-Holstein, Campus Lübeck, 23562 Lübeck, Germany; 11 Pathology, Research Center Borstel, Leibniz Lung Center, 23845 Borstel, Germany; 12 German Center for Lung Research (DZL), 35392 Gießen, Germany

**Keywords:** cisplatin, ifosfamide, paclitaxel, TIP combinational therapy, aggressive variant of prostate cancer, metastatic castration-resistant prostate cancer, DNA damage, DDR alteration

## Abstract

Significant progress has been achieved in the treatment of metastatic castration-resistant prostate cancer (mCRPC). However, results in patients with aggressive variant prostate cancer (AVPC) have been disappointing. Here, we report retrospectively collected data from intensively pretreated AVPC patients (n = 17; 88.2% visceral metastases; 82% elevation of neuroendocrine markers) treated with salvage chemotherapy consisting of cisplatin, ifosfamide, and paclitaxel (TIP). At the interim analysis, 60% of patients showed radiographic response or stable disease (PFS = 2.5 months; OS = 6 months). In men who responded to chemotherapy, an OS > 15 months was observed. Preclinical analyses confirmed the high activity of the TIP regimen, especially in docetaxel-resistant prostate cancer cells. This effect was primarily mediated by increased cisplatin sensitivity in the emergence of taxane resistance. Proteomic and functional analyses identified a lower DNA repair capacity and cell cycle machinery deficiency to be causative. In contrast, paclitaxel showed inconsistent effects, partially antagonizing cisplatin and ifosfamide in some AVPC models. Consequently, paclitaxel has been excluded from the TIP combination for future patients. In summary, we report for the first time the promising efficacy of TIP as salvage therapy in AVPC. Our preclinical data indicate a pivotal role for cisplatin in overcoming docetaxel resistance.

## 1. Introduction

In the past two decades, several life-prolonging therapies have been approved for the treatment of metastatic castration-resistant prostate cancer (mCRPC), e.g., abiraterone/prednisone, cabazitaxel (Caba), docetaxel (Doce), enzalutamide, and radium-223. In addition, inhibitors of Poly(ADP-Ribose)-Polymerase (PARP) recently became available for patients harboring DNA repair defects, and 177Lu-PSMA-617 radioligand therapy has been approved by the U.S. Food and Drug Administration (FDA) earlier this year. Despite this significant progress, the development of resistance and aggressive variants of this disease remain a major therapeutic challenge. In fact, men with visceral metastasis, particularly to the liver, face a dismal prognosis [1]. The same is true for men with neuroendocrine transdifferentiation. To better capture this group of patients, Aparicio and colleagues established seven criteria to identify men with aggressive variants of prostate cancer (AVPC, previously known as anaplastic prostate cancer) [2]. These include histologic evidence of small-cell prostate carcinoma (C1), exclusively visceral metastases (C2), radiographically predominant lytic bone metastases (C3), bulky (≥5 cm) lymphadenopathy, or bulky (≥5 cm) high-grade (Gleason ≥ 8) tumor mass in prostate/pelvis (C4), low PSA (≤10 ng/mL) at initial presentation (before ADT or at symptomatic progression in the castrate setting) plus high volume (≥20) bone metastases (C5), presence of neuroendocrine markers in histology or in serum at initial diagnosis or at progression plus any of the following in the absence of other causes (elevated serum LDH; malignant hypercalcemia, elevated serum CEA) (C6), short interval (≤6 months) to androgen-independent progression following the initiation of hormonal therapy with or without the presence of neuroendocrine markers (C7) (for detailed description, please review [2]). There is a general agreement that standard therapies often show insufficient effects in these patients. For this reason, we offered patients with signs of aggressive disease progression triple “TIP” chemotherapy consisting of cisplatin (Cis), ifosfamide (Ifo), and paclitaxel (Pac), a regimen that is primarily used as salvage therapy for germ cell tumors or penile carcinoma [3,4]. While, to the best of our knowledge, triple combination chemotherapy has not been used in mCRPC to date, the data on the three combination partners vary.

Platinum derivatives are alkylating and crosslinking substances showing moderate activity in an unselected mCRPC patients as mono- or combinational treatment [5,6]. However, an increasing evidence for a pronounced activity in AVPC and neuroendocrine differentiation has been reported [7]. Thus, the presence of at least one of the above-mentioned seven AVPC criteria has been associated with an increased likelihood of response to carboplatin and Doce, irrespective of morphology. In addition, in a randomized phase 2 trial the combination of carboplatin and Caba revealed a greater benefit in a pre-specified subgroup of men with clinical AVPC criteria compared with those without. Indeed, superiority of the carboplatin-based combination was even more pronounced if PTEN, RB1 and TP53 (molecular indicators of AVPC) were altered (≥2 genes detected by next generation sequencing or immunohistochemistry) [8]. Furthermore, increased activity has been reported in patients harboring DNA repair aberrations, particularly in *BRCA2* mutations carriers [5].

Taxanes inhibit mitosis by stabilizing microtubule but also AR nuclear translocation, reducing AR signaling. While Doce and Caba treatments have resulted in improved overall survival in phase 3 clinical trials, the development of paclitaxel (Pac) has not proceeded. Experience with Pac in men with Doce-refractory disease comes primarily from the combinational application with platinum compounds and/or estramustine phosphate [9,10,11]. Only moderate activity has been reported for the combination of carboplatin and Pac in unselected mCRPC patients in small patient cohorts, declining with each subsequent treatment line [12,13].

Ifo is an oxazophosphorine alkylating agent and interferes with DNA through formation of phosphotriesters and DNA-crosslinks, thereby inhibiting protein and DNA synthesis [14]. To date, data on the efficacy of Ifo combinations in mCRPC are sparse. A combination of Ifo and Doce was investigated in a phase 1/2 clinical trial revealing moderate activity with objective PSA response rates in 32% [15], however, significant toxicity was observed. Interestingly, in a Japanese patient cohort, promising efficacy of Cis and Ifo was reported in patients with advanced prostate cancer. Of note, these data were obtained in a time when Doce was not yet integrated as standard therapy in the treatment of mCRPC. For this reason, it may be assumed that mainly classical adenocarcinomas and not AVPC were evaluated [16].

Here, we report on the activity of a platinum-based combination with Ifo and Pac in heavily pretreated mCRPC patients harboring features of AVPC. Our preclinical data underscore the promising potential of this combination therapy, especially in the case of Doce-resistance induced by the long-term treatment.

## 2. Results

### 2.1. Patient Baseline Characteristics

A total of 17 patients were treated with combinational chemotherapy of Cis, Ifo and Pac at the University Hospital Hamburg-Eppendorf between 11/2013 and 09/2020 (Table 1). Median age at treatment initiation was 65 years ranging from 55 to 75 years. A total of 37% of the men were already metastatic at initial diagnosis. Patients were heavily pretreated with a median of four previous treatment lines (interquartile range (IQR) 3–6). All patients had previously received Doce and 65% of them also received Caba. In addition, the majority of the patients (82%) were treated with at least one new hormonal agent (NHA) i.e., abiraterone or enzalutamide. Other treatment regiments were applied in 41% of the men, including radium-223, cyclophosphamide, or carboplatin, in combination with Doce or etoposide.

At baseline, 15 patients (88.2%) suffered from visceral metastases, with the majority located in the liver (14 patients; 82%). Other visceral metastatic sites, including lung, adrenal gland, and brain, were present in eight men (47%). A total of 76% of the patients had bone metastases. PSA levels at baseline were highly variable, ranging from 0.01 ng/mL to 3110 ng/mL with a median of 77 ng/mL (IQR 10–640 ng/mL). Elevation of NSE was observed in 14 patients (82%). Median LDH was 903 IU/L (IQR 285–1728 IU/L), and alkaline phosphatase (AP) was 205 µg/l (IQR 131–440 µg/l) (Table 1). A total of 88.3% of the patients had at least one of the above-mentioned Aparicio criteria for AVPC, with most meeting C6 (12 of 17; 70.6%). Of the two patients who did not meet any of the criteria, one patient showed radiological progression 7 months after treatment initiation of ADT with novel pulmonary metastasis and a PSA < 1 ng/mL, while the other suffered from newly diagnosed hepatic metastases and intraspinal tumor progression negative for PSA-expression at the initiation of TIP treatment.

### 2.2. Dosage, Treatment Duration, and Discontinuation of Chemotherapy with TIP

At the median, three cycles of TIP were administered, with 29% of patients receiving all six cycles (Table 2). The median treatment duration was 3.5 months (IQR 1.4–3.8 months). In cycle 1, the median Pac dosage was 140 mg/sqm (IQR 132.5–162.5 mg/sqm), the median Ifo dosage was 2400 mg/sqm (IQR 2400–3200 mg/sqm), and the median cisplatin dosage was 40 mg/sqm (IQR 40–60 mg/sqm). In four patients, Cis was replaced by carboplatin AUC4 (day 1) due to contraindications (renal insufficiency, hearing loss, or peripheral neuropathy). Twelve patients (71%) discontinued treatment before completion of the six cycles of chemotherapy due to disease progression (8 patients) or adverse events (polyneuropathy (n = 1), infection (n = 1), or Ifo-induced psychosis (n = 2)) (Table 1).

### 2.3. Efficacy of TIP in mCRPC

**Figure 1:**
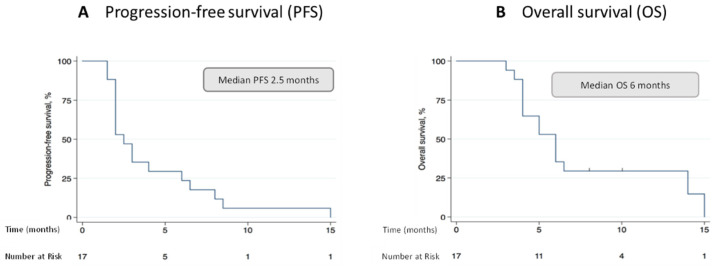
Kaplan–Meier curves showing progression-free survival (**A**) and overall survival (**B**) of mCRPC patients who received TIP therapy. Patients were treated at the University Cancer Center Hamburg-Eppendorf (UCCH). The data were analyzed retrospectively.

Radiological response was assessed after 2–3 cycles of chemotherapy and at the time of treatment discontinuation (Table 2). At interim analyses, 10 out of 17 patients showed radiological response or stable disease (58.8%), while 6 patients were primarily progressive (35.3%). One patient was not assessed due to infectious complications and denied further examinations. Five patients completed all six cycles of chemotherapy with two patients achieving a partial remission, one patient a stable disease, one patient a mixed response and one patient showed progressive disease at final analyses. Patients with increased baseline levels showed a ≥ 30%-PSA decline in 46% (6/13), a ≥ 50%-LDH reduction in 50% (8/16) and a NSE decrease in 62% (8/13) at any time during the course of therapy (Table 2). The median PFS was 2.5 months (Figure 1A), and the median OS was 6 months (Figure 1B).

### 2.4. Toxicity and Tolerability of TIP

Adverse events were recorded in all patients in the retrospective analyses of the data. These included worsening of general condition (n = 5), infectious complications including urinary tract infections (n = 5), decreased renal function or tubulopathy (n = 4), peripheral polyneuropathy (n = 3), and leukopenia (n = 2) or thrombocytopenia (n = 2). One patient had hydrope decompensation, and one patient showed signs of hepatotoxicity. Two patients developed tumor lysis syndrome after the application of chemotherapy. Four patients discontinued treatment due to adverse (see Section 2.2).

### 2.5. Cytotoxic Effects of the Individual Drugs and Their Combinations in PCa Cells Bearing Different Levels of Drug Resistance

**Figure 2:**
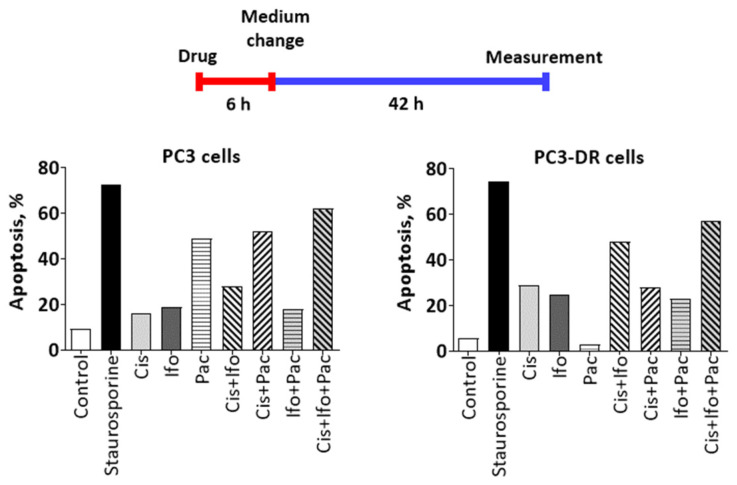
Effect of the drugs on caspase activity. Upper panel: Schematic representation for the experiment flow. PC3 or PC3-DR cells were treated with Cis, Ifo, and Pac at concentrations equal to their corresponding IC_50_s either individually or combined. The pan-caspase activity was measured after 48 h using FAMFLICA™ Poly Caspases Assay Kit. Cells treated with 1 µM staurosporine for 12 h were used as a positive control.

Given the promising clinical activity of the TIP combination in patients with AVPC, we decided to further elucidate the efficacy of each chemotherapeutic component. Therefore, cytotoxicity of each individual compound was determined in PCa cell lines harboring different levels of drug resistance, namely NHA-sensitive LNCaP cells (AR^+^), NHA-resistant 22Rv1 cells (AR^+^, AR-V7^+^), LASCPC-01 cells (NEPC), NHA-resistant and Doce-sensitive PC3 and DU145 cells (both are AR^-^) and their correspondent Doce-resistant counterparts PC3-DR and DU145-DR cells (Table 3). Expectedly, in comparison with PC3 and DU145 cells, the resistant PC3-DR and DU145-DR cells exhibited 20-40-fold resistance to Doce and 2-8-fold cross-resistance to Pac (Table 3). No specific cytotoxic activity for the Cis or Ifo was found in NHA-resistant cells (Table 3). Instead, Cis and Ifo showed increased cytotoxic effects (6-7-fold and 1.4-fold, respectively) in Doce-resistant PC3-DR and DU145-DR sublines compared to their sensitive counterparts PC3 and DU145. In line with this, apoptosis was increased in PC3-DR cells treated with Cis and Ifo at their IC_50s_ either individually or combined in comparison with the effect observed in PC3 cells (Figure 2). As expected, Pac failed to induce apoptosis in Doce-resistant cells, while it induced apoptosis profoundly in their sensitive counterparts.

Of note, both Cis and Ifo exhibited high activities in 22Rv1 (4.7 ± 1.06 µM and 4.8 ± 0.82 mM, respectively) and the NEPC LASCPC-01 (0.94 ± 0.06 µM and 3.13 ± 0.61 mM, respectively) cells (Table 3).

### 2.6. Cis as a Single Agent or in Combinations Is More Effective in Doce-Resistant PCa Cells

**Figure 3:**
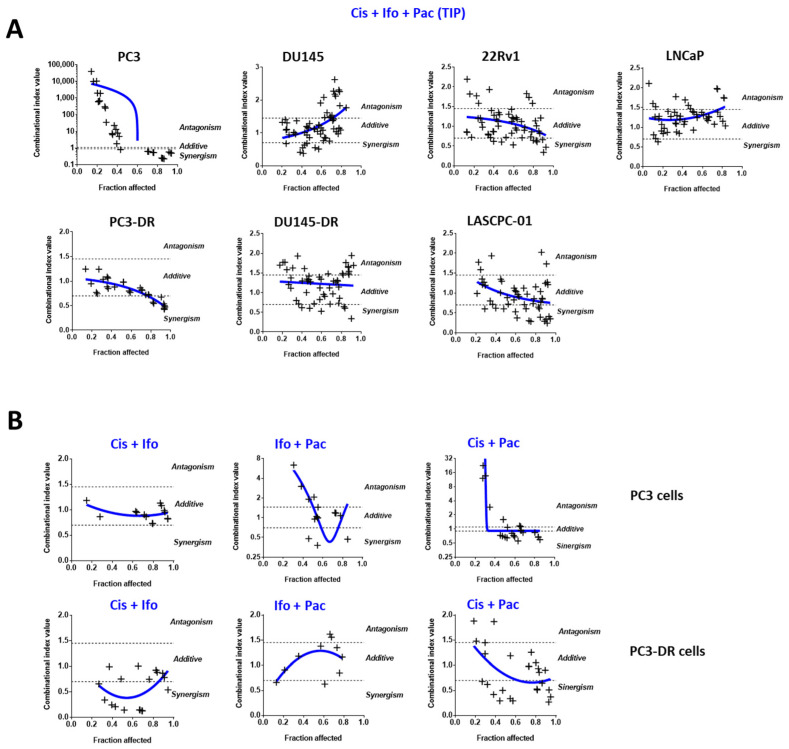
Cytotoxic effects of Cis, Ifo, and Pac and their combinations. The indicated prostate cancer cell lines were treated with the combination of three (**A**) or two (**B**) individual drugs for 48 h. Cell viability was evaluated using MTT assay and the data were further analyzed using Chou–Talalay method and CampuSyn 1.0 software. Effects of the drug combinations having certain combinational index (CI) were assumed as: synergistic, CI < 0.7; additive, CI = 0.7–1.45; antagonistic, CI > 1.45.

Next, we sought to further investigate the impact of individual drugs on the anticancer effect of the TIP combination. For this, the single agents were combined at the ratios of their corresponding IC_50_s (see Table 3), and the potential synergistic effects were evaluated using an MTT assay and a Chou–Talalay method (Figure 3A). The Chou–Talalay method for drug combination applies the theoretical foundation for the combinational index (CI) and the isobologram equation, which enables the use of the CompuSyn software to quantitatively determine medication interactions. In this approach, CI < 0.75, 0.75–1.45, and > 1.45 represent synergistic, additive, and antagonistic effects, respectively. Our results revealed additive to synergistic effects of the TIP combination in 22Rv1, LASCPC-01, and LNCaP cells (Figure 3A). Remarkably, antagonistic effects were observed in the AR-negative PC3 and DU145 cells treated with the TIP combination, while in the correspondent Doce-resistant clones, the combination exhibited additive to synergistic effects (Figure 3A). In line with this, higher cleavage of caspase-3 and PARP (apoptotic markers) was detected in the PC3-DR and 22Rv1 cells treated with the TIP combination when compared to the cells treated with the single drugs (Figure S7). To further assess the contribution of the individual agents in the context of TIP therapy, combinational treatment with two of the three drugs was performed, and cytotoxic effects were analyzed in PC3 and their Doce-resistant PC3-DR subline (Figure 3B). In PC3-DR cells, all two-drug combinations revealed additive to synergistic effects, especially if Cis was included (Figure 3B). In contrast, in PC3 cells, a strong antagonistic effect was detected at low concentrations of Pac combined with either Cis or Ifo (Figure 3B). Taken together, these data suggest that Cis is beneficial for the treatment of Doce-resistant PCa cells, while Pac shows rather inconsistent results.

### 2.7. Differential Effects of TIP on the Proteome of Doce-Sensitive and -Resistant PCa Cells

To obtain more insights into the molecular mechanism of TIP activity in Doce-resistant cells we firstly compared the changes in the global proteome profiling of PC3 and PC3-DR cells. In total, 4546 proteins were identified and quantified across all samples (Table S1). Comparative analysis of the proteomic profiles of the two cell lines (Table S2) revealed a significant enrichment of ribosomal RNA (rRNA) processing processes in the drug-resistant PC3-DR cells (Table S7), indicating an accelerated rRNA processing and ribosomal biogenesis, probably to cover a higher protein demand required for increased cell growths [17]. In line with this finding, a concomitant increase in the proliferation marker Ki-67 was observed in PC3-DR cells (Table S1). Moreover, consistent with the Doce-resistant nature of PC3-DR cells, a higher expression of p-glycoprotein (p-gp, ABCB1) (Table S1), negative regulation of pro-apoptotic cytochrome C release from mitochondria as well as positive regulation of tubulin polymerization and depolymerization were detected in these cells (Table S8). Finally, PC3-DR cells showed an elevated level of β-catenin (catenin beta 1; CTNNB1), suggesting an upregulation of canonical Wnt/β-catenin signaling in the drug-resistant cells (Table S1).

**Figure 4:**
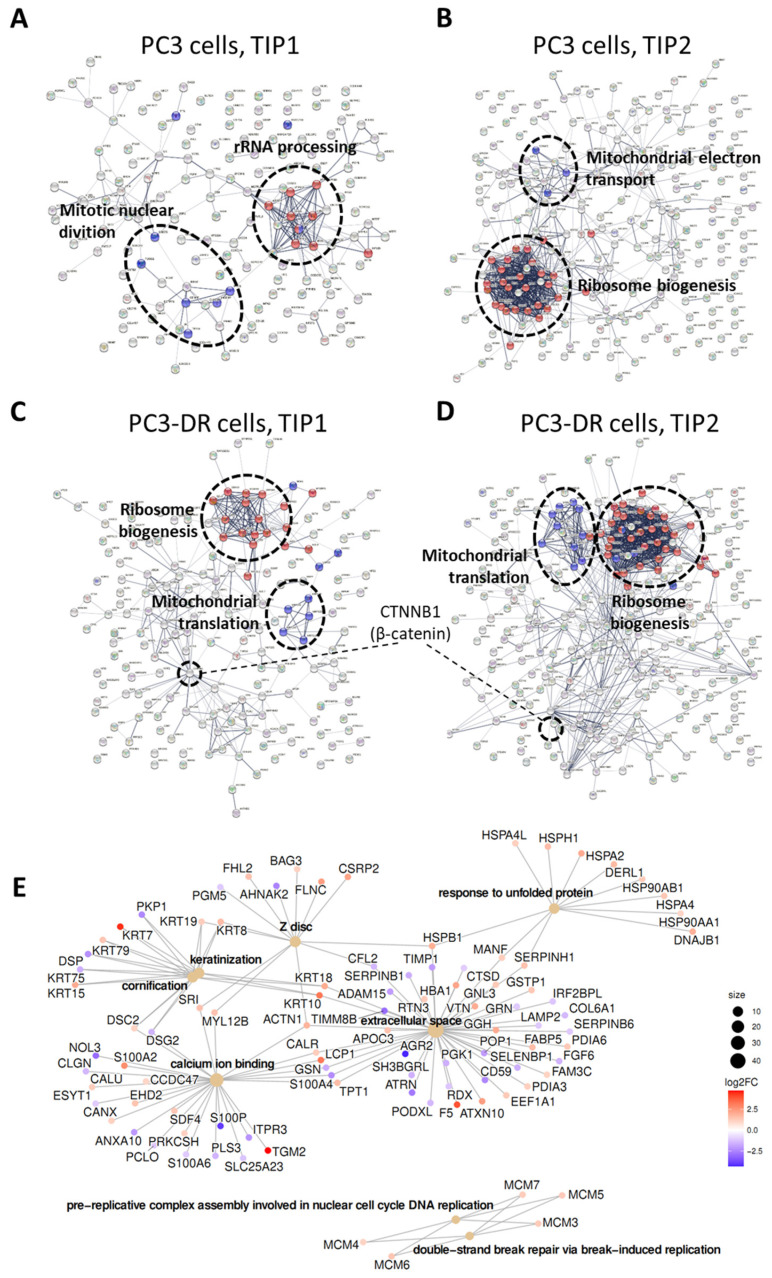
Protein network analysis and protein clustering. PC3 (**A**,**B**) or PC3-DR (**C**,**D**) cells were treated with TIP1 (**A**,**C**) or TIP2 (**B**,**D**) (see Materials and Methods) combination for 48 h, and the changes in proteome were identified using LC-MS/MS technique. Further functional enrichments, gene ontology, as well as the construction of the hypothetical protein interaction networks were performed using STRING v.11.5 software. The proteins used for the analysis are identical to those listed in Tables S3–S6. (**E**), Interaction network of significantly differentially expressed proteins in PC3 cells versus PC3-DR cells, both treated with TIP2 combination, that is associated with significantly enriched GO terms performed using the Molecular Signatures and the Gene Ontology databases.

Next, we compared the TIP-induced changes in the proteomic profiles of PC3 and PC3-DR cells. Cells were treated with two different drug combination concentrations, namely, TIP1 or TIP2 (see Materials and Methods). In total, 153 (TIP1, Table S3) and 234 (TIP2, Table S4) proteins in PC3 cells versus 179 (TIP1, Table S5) and 257 (TIP2, Table S6) proteins in PC3-DR cells were detected to be significantly regulated by the treatment. A protein interaction network analysis was generated by STRING (Figure 4) for our comparative analysis of the TIP-induced differences in protein profiles between the two cell lines. TIP induced significant changes in RNA processing as well as ribosomal- and mitochondria-related processes in both cell lines (Figure 4A–D, Tables S9–S12), indicating suppression of cancer cell metabolism.

In Doce-sensitive PC3 cells, TIP was also predicted to influence mitotic nuclear division (Table S9), suggesting a drug-induced effect on cell cycle progression. In contrast, we found a downregulation of proliferation marker Ki-67 as well as a downregulation/degradation of different histones in TIP-treated PC3-DR cells. Interestingly, several DNA double-strand break (DSB) repair proteins such as Rad21 (RAD21), Ku80 (XRCC5), Ku70 (XRCC6), PARP1 (PARP1), and MRE11 (MRE11) were found to be downregulated (Table S1). Although the difference for some of the proteins failed to reach significance, these data reveal a possible role for DNA DSB repair in the cellular response to TIP treatment. In addition, decreased enrichment of integrins and cadherins (essential for cancer cell migration, adhesion, and angiogenesis, Tables S11 and S12) were detected secondary to TIP exposure (Tables S5 and S6). Finally, although we suggested activation of canonical Wnt/β-catenin signaling in the drug-resistant cells, TIP was found to inhibit the β-catenin in the Doce-resistant cells, suggesting an inhibition of the canonical Wnt/β-catenin signaling by TIP (Tables S1, S5 and S6).

Further bioinformatical analysis of the overrepresented pathways was performed using Molecular Signatures and the Gene Ontology databases. It revealed two significantly enriched GO terms, “Regulation of pre-replicative complex assembly involved in nuclear cell cycle DNA replication (GO:0006267)” and “Double-strand break repair via break-induced replication (GO:0000727)” in PC3 cells compared with PC3-DR cells treated with the TIP combination (Figure 4E, Table S13), suggesting different effects of TIP treatment on cell cycle as well as DNA damage and repair processes in the context of Doce-resistance development.

In conclusion, proteomics data proposed a cell cycle arrest in PC3 cells and induction of apoptosis in PC3-DR cells secondary to TIP therapy. The latter event was attributed to disruption of DNA-reparation system.

### 2.8. Validation of the Proteomics Data

Validation of the proteome analysis data revealed significantly elevated levels of both N- and E-cadherins, as well as anti-apoptotic protein survivin in PC3-DR cells compared to PC3 cells, consistent with the increased aggressiveness and therapy resistance of the PC3-DR cells (Figure S8). The expression of stem cell markers Sox-2, Oct-4, enolase 2, and neuroendocrine markers chromogranin and synaptophysin were similar in both cell lines, indicating that no cell rewiring in terms of stemness or neuroendocrine transformation was involved (Figure S8). Moreover, while the total Rb protein level was rather comparable in both PC3 and PC3-DR cells, the level of its inactive phosphorylated form was upregulated in PC3-DR cells, suggesting different cell cycle regulation in both cell lines.

Consistent with the results of the proteomic analyses, we confirmed the TIP-induced downregulation of β-catenin in PC3-DR using Western blotting analysis (Figure S9). Additionally, a dose-dependent-induced downregulation of both N- and E-cadherins was observed in PC3-DR cells (Figure S9). Furthermore, PARP1 was found to be downregulated under TIP treatment (Figure S9). The suggested decrease in PARP1 could be explained by the increased PARP cleavage observed in PC3-DR cells (Figure S7), which was well correlated with induction of apoptosis (Figure 2) and downregulation of survivin (Figure S9).

**Figure 5:**
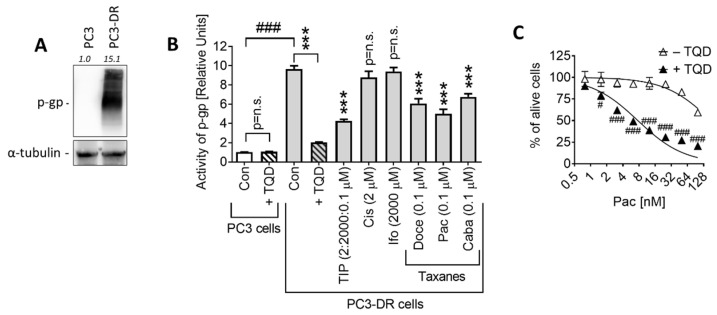
P-gp expression and activity. (**A**), p-gp expression in PC3 and PC3-DR cells was evaluated with Western blotting. (**B**), Effect of the investigated drugs and TIP combinations on the activity of p-gp in PC3-DR cells measured using calcein-AM efflux assay. The observed drop-down of p-gp activity indicates the efflux of the drugs via the p-gp system. (**C**), Viability of PC3-DR cells treated with Pac for 48 h with and without pre-treatment (1 h) with 50 nM tariquidar (TQD, p-gp inhibitor). Cell viability was evaluated using an MTT assay. Statistical analysis: difference between the sample and control: *** *p* < 0.001 (ANOVA); difference between two samples: ^#^ *p* < 0.05; ^###^ *p* < 0.001 (*t*-test).

A suggested overexpression of p-glycoprotein (p-gp; MDR1) in PC3-DR cells was further verified by Western blotting (Figure 5A). In addition, p-gp activity was measured by a calcein-based assay. Calcein (calcein-AM) is a dye, which is excreted from the cells via p-gp [18]. If the activity of p-gp is decreased, the dye accumulates in the cells and undergoes conversion to green-fluorescent derivative, which is driven by intracellular esterases. Indeed, we observed that both, well-established inhibitor tariquidar (TQD) and taxanes (known p-gp substrates) inhibited the p-gp-driven calcein-AM efflux (Figure 5B). In consistence with the increased p-gp level in PC3-DR cells, a significantly higher activity of p-gp was detected in Doce-resistant PC3-DR cells compared to their Doce-sensitive counterparts (Figure 5B).

Pac and Doce are known substrates of p-glycoprotein. Thus, increased expression and activity of p-glycoprotein lead to greater elimination of both chemotherapeutic agents from the tumor cells and, consequently, to a reduction in their cytotoxic activity [19]. In contrast, Caba has been designed to have a lower affinity to p-gp and shows minor cross-resistance with other taxanes [19]. In line with this, the concurrent inhibition of calcein efflux was most pronounced by Pac, followed by Doce and Caba (Figure 5B), reflecting the affinity of these drugs to p-gp. An inhibition of p-gp by TQD expectedly resulted in a dramatic increase in Pac cytotoxic activity on PC3-DR cells (Figure 5C). Importantly, neither Cis nor Ifo affected p-gp activity (Figure 5B), suggesting no or minor efflux of the two cytotoxic agents in docetaxel-resistant cells. Hence, the concurrent inhibition of calcein efflux observed under TIP administration should be exclusively induced by Pac.

### 2.9. Defective DNA Repair Mechanisms in Doce-Resistant Cells Result in Higher Therapy Efficacy

**Figure 6:**
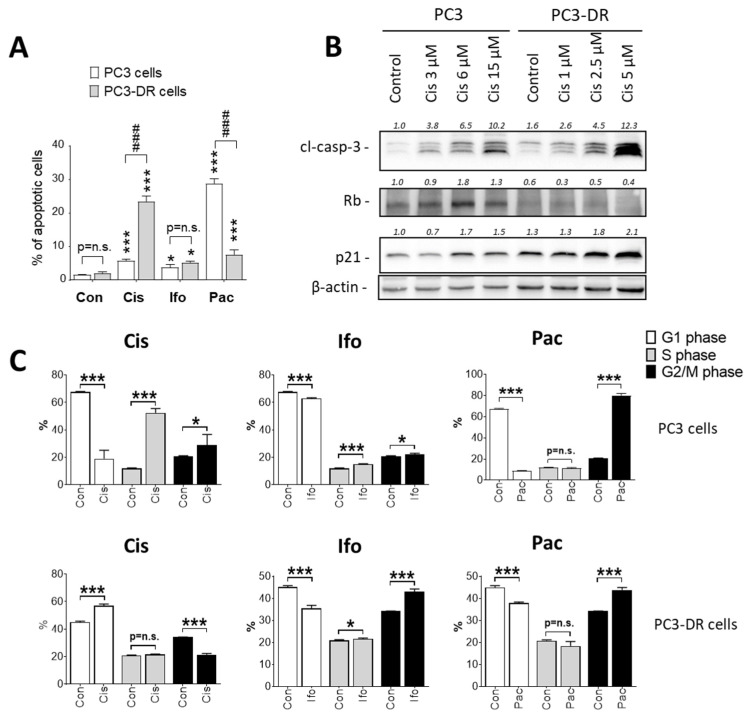
Effects of the individual drugs on cell cycle progression. DNA fragmentation (**A**) and cell cycle progression (**C**) in PC3 and PC3-DR cells were accessed using flow cytometry technique and propidium iodide (PI) staining. Cells were treated with Cis (10 µM), Ifo (5 mM), or Pac (250 nM) for 48 h, harvested, fixed, stained with PI, and analyzed. Cells that appeared in the sub-G1 population were assumed as apoptotic (**A**). (**B**), Protein expression was measured in the drug-treated cells using Western blotting. In all the experiments, cells were treated with the drugs for 48 h. Statistical analysis: difference between the sample and control: * *p* < 0.05; *** *p* < 0.001 (ANOVA); difference between two samples: ^###^ *p* < 0.001 (*t*-test).

The combined data of cytotoxicity analysis, drug combinational studies, and proteome analysis suggested Cis to be primarily responsible for the activity of TIP in the Doce-resistant PC3-DR cells. This was attributed to a more effective induction of apoptosis (Figure 6A,B) and a less effective DNA repair system in PC3-DR cells.

Proteomics data also suggested defective DNA repair pathways to be involved in higher sensitivity of Doce-resistant cells to Cis. Following DNA damage, the cells use their checkpoints to delay cell cycle in order to spare time for DNA repair. Impaired checkpoints or repair will cause replication stress and apoptosis. Indeed, cell cycle analyses revealed a pronounced S- and G2/M-arrest in PC3 cells treated with Cis (Figure 6C). On the contrary, PC3-DR cells exposed to the same concentration of Cis exhibited no such arrest, instead a G1-arrest was detected (Figure 6C). In line with this, we found a dose-dependent upregulation of Rb (associated with both, cell cycle arrest and DSB repair [20]) in Cis-treated PC3 cells, while no such effect was observed in PC3-DR cells (Figure 6B). Although p21 was upregulated in both cell lines, a higher dose-dependent increase in p21 expression was reported in PC3-DR compared to PC3 cells (Figure 6B). This observation was in line with the increased G1-arrest in PC3-DR cells.

**Figure 7:**
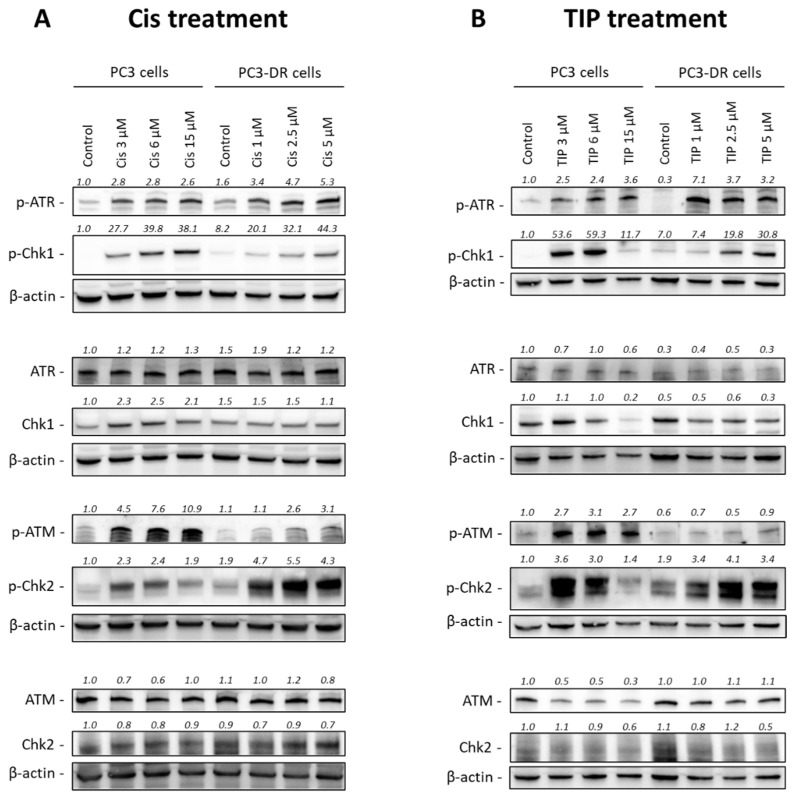
Effect of Cis and TIP on the protein expression. The level of protein expression was analyzed by Western blotting. PC3 or PC3-DR cells were treated with indicated concentrations of Cis (**A**) or TIP (**B**) for 48 h. For TIP treatment the following drug concentrations were used: for PC3 cells TIP 3 (Cis 3 µM, Ifo 2 mM, Pac 30 nM), TIP 6 (Cis 6 µM, Ifo 4 mM, Pac 60 nM), TIP 15 (Cis 15 µM, Ifo 10 mM, Pac 150 nM); for PC3-DR cells TIP 0.5 (Cis 0.5 µM, Ifo 2 mM, Pac 50 nM), TIP 1 (Cis 1 µM, Ifo 4 mM, Pac 100 nM), TIP 2.5 (Cis 2.5 µM, Ifo 10 mM, Pac 250 nM).

In consistence with the different regulation of the cell cycle in both cell lines, we observed an activation of both ATR/Chk1 and ATM/Chk2 axes in PC3 cells treated with either Cis as a single agent (Figure 7A) or the TIP combination (Figure 7B). On the contrary, either treatment resulted in no or weak Chk1 activation PC3-DR cells, while Chk2 was over-activated, probably as a compensatory mechanism for the weak Chk1 signaling (Figure 7A,B). Chk1 is known to be activated in response to replication stress, leading to S- and G2/M-arrest [21]. On the other hand, Chk2 is often activated as a result of DSBs and leads to G1-arrest to prevent entering S-phase with damaged DNA [21].

**Figure 8:**
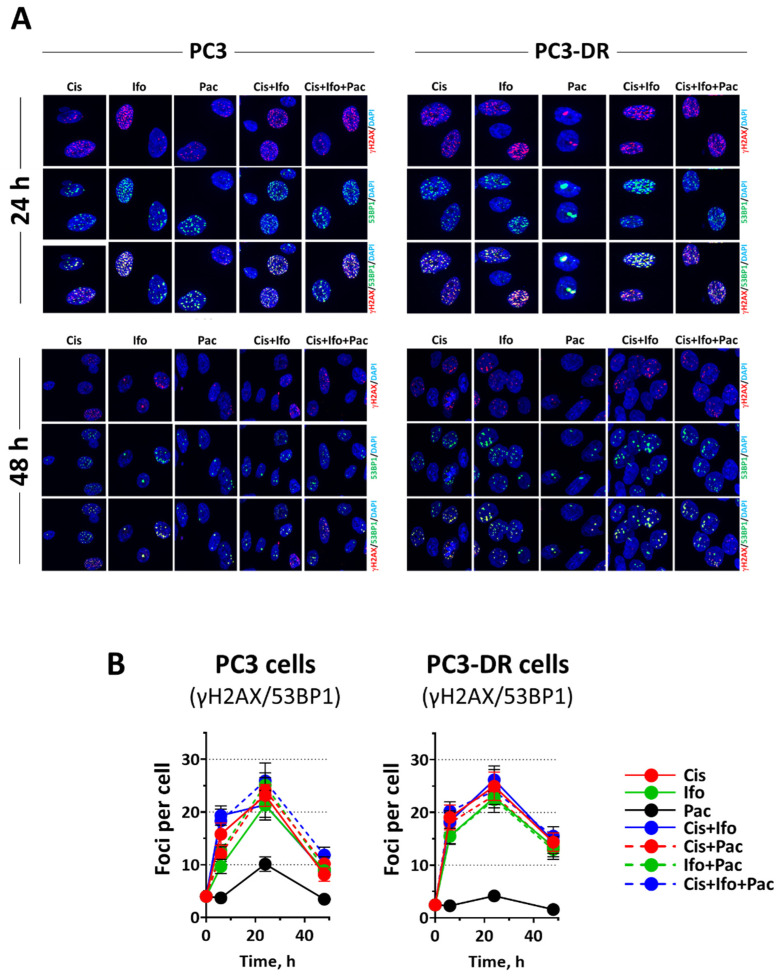
Evaluation of DNA double-strand breaks (DSB) in the cells following the treatment. PC3 and PC3-DR cells were treated with the individual drugs at concentrations equal to their corresponding IC_50_s and their combinations for 6 h. (**A**), 6 h, 24 h, and 48 h following the treatment, immunofluorescence double staining for γH2AX (red) and 53BP1 (green) was performed. (**B**), Colocalized γH2AX/53BP1 foci were considered as a marker of DSB and were quantified microscopically. For each experimental condition, at least 100 cells were analyzed. Data are indicated as means ± SD from at least three independent experiments. The original data are presented in Figure S10 and Table S14.

It is plausible then to assume that increased G1-arrest and the defective S/G2/M checkpoints indicate an impaired DNA repair to be less effective in the Doce-resistant PC3-DR cells compared to their Doce-sensitive counterparts. In order to address this assumption, both PC3 and PC3-DR cells were treated with the IC_50_ concentrations of Cis, Ifo, and Pac either individually or combined, then DSB repair was investigated by monitoring the well-established DSB-markers γH2AX and 53BP1 at 6 h, 24 h, and 48 h post-treatment (Figure 8A,B and Figure S10).

**Figure 9:**
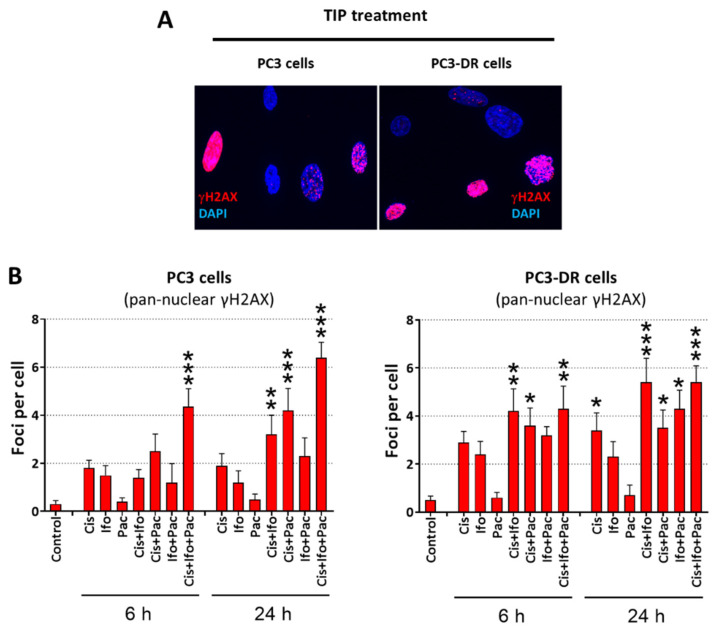
Evaluation of cells undergoing replication stress following the treatment. PC3 and PC3-DR cells were treated with the individual drugs at concentrations equal to their corresponding IC_50_s and their combinations for 6 h. A total of 6 h and 24 h following the treatment immunofluorescence staining for γH2AX (red staining) and nuclei (DAPI, blue staining) was performed (**A**). Cells exhibiting a pan-nuclear γH2AX signal were considered as those undergoing replication stress and were microscopically counted (**B**). Statistical analysis: difference between the sample and control: * *p* < 0.05; ** *p* < 0.01; *** *p* < 0.001 (ANOVA).

Interestingly, although both cell lines showed a treatment-induced increase in pan-nuclear γH2AX staining, the increase was more pronounced in PC3-DR cells after treatment by the three cytotoxic drugs as well as their combinations (Figure 9A,B), indicating increased drug-induced replication stress in PC3-DR cells. Of note, the replication stress induced by TIP combination in PC3 cells was higher in comparison with the stress induced by any other two-drug combinations, whereas in PC3-DR cells the efficacy of TIP was comparable to the Cis + Ifo combination (Figure 9A,B).

In keeping with our hypothesis that PC3-DR cells exhibit less effective repair capacity, we revealed a higher number of γH2AX/53BP1 foci at 6 h post-treatment with Cis and Ifo either individually or combined. No difference in the number of DSB foci was observed at 24 h post-treatment time point with Cis or Ifo in single or combined regimen. Importantly, after 48 h post-treatment, we demonstrated a significantly higher number of γH2AX/53BP1 foci in PC3-DR cells after single (*p* = 0.002 and *p* = 0.048, for Cis and Ifo, respectively) or combined treatment (*p* = 0.003), emphasizing lower DNA repair efficiency in Doce-resistant PC3-DR cells. This is in agreement with the observed higher replication stress in the drug-resistant cells under the treatment (Figure 9). Notably, even though in TIP regimen the number of residual DSB foci at the 48 h time point was higher in PC3-DR cells, no significant difference with PC3 cells was observed (*p* = 0.13). Collectively, these data reflect therefore the higher efficacy of Cis in PC3-DR cells due to the defective/less effective DNA repair system.

## 3. Discussion

Platinum-based combinations with Doce and Caba have shown promising results in AVPC with or without neuroendocrine transdifferentiation, whereas in pure neuroendocrine prostate carcinomas, a platinum-etoposide combination can be considered as standard therapy [22]. However, aggressive transdifferentiation of prostate cancers often occurs late in treatment, so a considerable number of patients have already received therapy with both approved taxanes. In addition, treatment alternatives are lacking after the failure of the platinum-etoposide combination in NEPC. Moreover, the optimal therapeutic strategy in “double-negative” patients with a loss of typical adenocarcinoma differentiation but without concurrent neuroendocrine markers remains elusive [23]. Thus, there is a high medical need for novel treatment approaches.

In this setting, the combination of Cis, Ifo, and Pac might be a promising new option. In our current retrospective analysis of AVPC patients, 60% of the men achieved an at least stable radiological disease at interim analyses and nearly 30% were able to receive all six cycles of chemotherapy. The median OS was 6 months, and overall survival of > 15 months was achieved in patients who responded to chemotherapy. Of note, the vast majority of the patients suffered from visceral metastasis, mostly located in the liver, and had received a median of four previous treatment lines, including Doce in all and Caba in two-thirds of the cases. Despite these very unfavorable prognostic features, disease control and prolonged survival was achieved by the TIP regimen.

In addition, we were able to confirm our promising retrospective clinical results using different in vitro PCa cell models reflecting the AVPC of our patients. In line with the clinical data, TIP exhibited an additive to synergistic effect in NHA-resistant and Doce-resistant cell lines as well as in NEPC. Of note, AR-negative cells were less responsive to TIP, while their Doce-resistant counterparts exhibited an increased sensitivity. This has been found to be mainly attributed to acquired mechanisms resulting in pronounced cellular sensitivity to Cis during the development of Doce-resistance. Further analysis revealed that Cis and to lesser extend Ifo treatment induces efficient S- and G2/M arrests in Doce-sensitive cells by activation of ATR/Chk1 checkpoint, which indeed spares time for repair of Cis-induced DNA damage. However, Doce-resistant cells failed to activate S- or G2/M checkpoints, instead a mild G1-phase arrest was observed. Consistent with this, a less effective DNA repair was reported in PC3-DR cells compared to their Doce-sensitive counterparts as evidenced by (i) an increased number of residual γH2AX/53BP1 foci, (ii) a higher pan-nuclear γH2AX staining (a marker for replication stress), and (iii) enhanced apoptosis level in PC3-DR after treatment with the IC_50_s of Cis and Ifo either individually or combined. Taxane-induced sensitization of cancer to platinum agents has been previously reported [2,24]. This effect was recently identified to be at least partly caused by the inhibition of the CXCR2/BCL-2 axis [25]. However, the precise mechanism has not been clarified to date. In our experiments, pronounced activity of TIP was observed in the Doce-resistant PC3-DR and DU145-DR. Both cell lines are constantly cultured in the medium containing Doce. This in fact imitates the in vivo situation, as all patients included in our analyses were heavily pretreated with Doce as well. In the global proteome screening analyses, PC3-DR cells revealed typical signs of taxane resistance, e.g., upregulation of p-glycoprotein, suppression of intrinsic apoptotic pathway (i.e., negative regulation of pro-apoptotic cytochrome C release from mitochondria and positive regulation of survivin), accelerated protein metabolism and promotion of tubulin polymerization/depolymerization. In line with previously reported data by de Porras et al. [25], the pro-apoptotic effects of TIP in taxane-resistant cells were stipulated by downregulation of Ki-67, suppression of ribosomal function and inhibition of several DNA repair proteins. Thus, it is plausible to speculate based on these data that long-term exposure to taxanes and formation of resistance may lead to the inhibition of repair of the Cis-induced DNA damage by alteration of expression of the corresponding genes. We and others found that Cis cytotoxicity is promoted by reduced repair of DSB followed by apoptosis due to overwhelming DNA damage [26]. In line with this, in taxane-resistant cells a quicker DNA damage and presumably lower efficacy of DSB repair following Cis or TIP treatment were shown. In addition, a defective cell cycle machinery (which was indicted by overphosphorylation of Rb, lack of non-phospho-Rb upregulation in response to cytotoxic agents, misregulation of ATR/Chk1 and ATM/Chk2 pathways) maybe causative for the increased apoptosis in Doce-resistant cells [27]. Interestingly, β-catenin, a downstream effector of the Wnt pathway was found to be downregulated in Doce-resistant PC3-DR cells under TIP treatment. Of note, the Wnt signaling pathway is known to be a major regulator of development and stemness in various solid tumors and induces the expression of NE-markers in PCa cells [28].

The contribution of Pac to the efficacy of TIP therapy appears to be much more inconsistent. An antagonistic effect on Cis and Ifo activity was found in some taxane-naïve, AR-negative PCa sublines. In addition, increased cellular efflux of Pac was observed, especially in Doce-resistant cell lines, while Cis and Ifo were not the substrates of drug resistance-mediating p-glycoprotein. This is in line with our previously described findings of p-glycoprotein upregulation secondary to long-term Doce exposure. Therefore, based on our data, the contribution of Pac to the efficacy of the triple combination is not clearly established and must be questioned. Further investigation is needed to address this issue.

Side effects posed a challenge in our intensively pretreated patients with mostly very advanced disease, although substantial dose reductions were made compared with the protocol originally used in germ cell tumor patients. In fact, for all patients, adverse events were documented, including infectious complications, PNP, and worsening of renal function. In total, four patients discontinued treatment early due to PNP, infection, and ifosfamide-induced psychosis. Here, sparing the additional toxicity of Pac may potentially reduce hematotoxicity and neurotoxicity and thus could make the treatment available to a larger cohort of patients with improved tolerability.

Limitations of our data are the retrospective study design and a long recruitment period with substantial changes in the treatment of metastatic prostate cancer. Due to limited availability, only the tumor tissue of a few patients underwent molecular testing. However, due to legal regulations, no further testing can be performed. Thus, we cannot state the impact of pre-existing DNA damage repair defects or gene alterations associated with transdifferentiation (e.g., *RB1*, *TP53*, *PTEN*) for treatment efficacy.

Of course, our data have to be evaluated against the background of the new treatment options for mCRPC. However, it must be noted, that in the majority of registration trials as well as in studies currently carried out with novel treatment approaches, patients with visceral metastases are often only small subgroups, while in our retrospective analyses 88% suffered from visceral metastases mostly located in the liver. Therefore, it is difficult to compare our results with other studies. PARP inhibition is a targeted treatment approach showing high activity especially in mCRPC patients harboring pathogenic BRCA1/2 alterations [29]. Due to the very limited molecular diagnostics available in our retrospective patient cohort, we cannot make a reliable statement on how many of our patients would have been amenable to PARP inhibition. However, pathogenic HRR alterations are present in only 20–30% of mCRPC patients [29]. Radioligand therapy, is promising newly approved therapy, however with decreased efficacy in patients with visceral disease [30,31]. In addition, patients with aggressive or neuroendocrine transdifferentiation often have lower PSMA expression and thus have limited access to this treatment. Immunotherapeutic treatment approaches have so far fallen short of the high expectations placed in them [32]. The first promising data for patients with visceral metastasis were now demonstrated for a combination with cabozantinib and atezolizumab. Remarkably, a high disease control rate was observed in patients with visceral and/or nodal disease outside the pelvis [33]. However, these patients were chemotherapy-naïve and thus do not correspond to the patient population we studied. BiTEs and CAR-T cells against, e.g., DLL3 and KLK2 are in early clinical testing. These targets are presumably also expressed at sufficient levels in the patient population described here, but results are still pending. A targetable, frequently activated pathway in mCRPC is the PI3K-AKT-mTOR pathway. To date, different targeted approaches have been developed to reverse activation. For example, Akt inhibitor ipatasertib has been examined in combination with abiraterone/prednisone in the phase 3 IPATential in chemotherapy-naïve patients [34]. Despite promising results, the mechanism of resistance to PI3K-AKT-mTOR pathway-targeted therapies is expected to vary dramatically between patients and within individual tumors due to a varying activity status of the pathway components, the extent of intratumoral heterogeneity, the mode and concentration of upstream stimuli, the genetic alterations present and the composition of the tumor microenvironment [35]. In addition, to the best of our knowledge no data are available on AVPC patients. A patient population that may be the most comparable to ours was studied by Beltran and colleagues in a phase 2 trial with the aurora kinase A inhibitor alistertib. Here, 60% of patients had hepatic metastasis (compared with 82% of patients in our study). Doce and Caba pre-therapy had been received by 32% and 10% of patients, respectively, compared with 100% and 65% in our cohort. The median PFS for this less intensively pretreated patient cohort was 2.2 months, and stable disease was observed at interim staging in 30% of patients [36]. In our very intensively pretreated retrospectively evaluated patient cohort, PFS was 2.5 months, stable disease or treatment response was achieved in 60% of patients at interim staging. This highlights the challenge in the treatment of AVPC patients and the continuing high unmet medical need for these patients.

## 4. Material and Methods

### 4.1. Clinical Study Design, Participants, and Experimental Treatment Intervention

Retrospective analyses were carried out for mCRPC patients who were treated with salvage chemotherapy with Cis/carboplatin, Ifo, and Pac in the Department of Oncology at the University Cancer Center Hamburg-Eppendorf (UCCH, Hamburg, Germany) between November 2013 and September 2020. Patients had progressed on all available standard therapies at the time and received in-depth clarification on the individualized treatment approach. In patients unsuitable for Cis due to impaired renal function, pre-existing polyneuropathy, or clinically relevant hearing impairment, carboplatin was applied instead. The dosage of the medication was adjusted to the general condition of the patients. The administration of Cis and Ifo was divided over 2 to 3 days, while Pac was applied as a single dose on day 1. Patients received G-CSF to prevent or reduce neutropenia based on personal needs.

### 4.2. End Points of the Clinical Study

Progression-free survival (PFS) and overall survival (OS) were determined to assess the effectiveness of chemotherapy. PFS was defined as the time from the administration of the first chemotherapy to radiological or serological progression or patient death, whichever occurred first. Radiological as well as serological responses were documented. To assess serological response, tumor markers PSA and NSE, as well as AP and LDH, were recorded.

### 4.3. Reagents

The following reagents were used: RNase (Carl Roth, Karlsruhe, Germany); cisplatin, ifosfamide, paclitaxel, docetaxel, and cabazitaxel (Pharmacy of the University Hospital Hamburg-Eppendorf, Hamburg, Germany); tariquidar (TQD; MedChemExpress, Monmouth Junction, NJ, USA); propidium iodide (PI) and MTT (3-(4,5-dimethylthiazol-2-yl)-2,5-diphenyltetrazolium bromide) (Sigma, Taufkirchen, Germany), RNase (Carl Roth, Karlsruhe, Germany). The antibodies used are listed in Supplementary File S1.

### 4.4. Cell Lines and Culture Conditions

Human prostate cancer cell lines PC3, DU145, 22Rv1, LNCaP, and LASCPC-01 were purchased from ATCC (Manassas, VA, USA). The Doce-resistant PC3-DR and DU145-DR cell lines, generated by the long-term exposure to increasing sub-lethal concentration of Doce as previously described [37], were donated by Prof. Z. Culig, Innsbruck Medical University, Austria. All cell lines were recently authenticated and cultured according to the manufacturers’ protocols.

### 4.5. In Vitro Cytotoxicity Assay (MTT Test)

The cytotoxic activity of the drugs was evaluated using an MTT assay as previously described [38]. Briefly, 6000 cells/well were seeded in a 96-well plate and were allowed to attach for 24 h before being treated with the indicated drugs for 48 h. MTT was then added in each well and incubated at 37 °C for 3 h to generate the formazon, which was dissolved in DMSO, and the resulting purple color was measured at 550 nm with the background reference measured at 655 nm in a plate reader Infinite F200PRO (TECAN, Männedorf, Switzerland).

### 4.6. Immunofluorescence

Treated cells on coverslips were washed once with cold PBS and fixed with 4% paraformaldehyde/PBS for 10 min. Fixed cells were permeabilized with 0.2% Triton X-100/PBS on ice for 5 min and incubated for 1 h at room temperature with primary anti-phospho-S139-H2AX antibody and anti-53BP1 antibody. After being washed three times with cold PBS, the cells were incubated for 1 h with secondary anti-mouse Alexa-fluor594 antibody or anti-rabbit Alexa-fluor488 antibody. The nuclei were counterstained with 4′-6-diamidino-2-phenylindole (DAPI, 10 ng/mL). Slides were mounted in Vectashield mounting medium (Vector Laboratories, Newark, CA, USA). Fluorescence microscopy was performed using the Zeiss AxioObserver.Z1 microscope (objectives: ×20, resolution 0.44 µm; Plan Apo 63/1.4 Oil DICII, resolution 0.24 µm; and filters: Zeiss 43, Zeiss 38, Zeiss 49) (Carl Zeiss, Göttingen, Germany). Z-stacks of semi-confocal images were obtained using the Zeiss Apotome, Zeiss AxioCam MRm, and Zeiss AxioVision Software (Carl Zeiss, Göttingen, Germany). DSBs were quantified using ImageJ and DAPI-based image masks and normalized to single nucleus values.

### 4.7. Caspase Activity Assay

Apoptosis was investigated by detection of caspase activity utilizing the FAMFLICA ™ Poly Caspases Assay Kit (Immunochemistry Technologies, Davis, CA, USA) according to the manufacturer’s instructions. Flow cytometric analysis was performed on a FACS Canto with FACS Diva Software (BD Bioscience, San Jose, CA, USA). Staurosporine (Sigma) was used as a positive control with a final concentration of 1 µM for at least 12 h incubation.

### 4.8. p-gp-Driven Efflux of Calcein-AM

An evaluation of p-gp activity was performed using calcein-AM dye (BIOZOL, Eching, Germany). The cells were seeded in black 96-well plates with transparent bottoms (10 × 10^3^ cells/well in 100 µL/well) and incubated overnight. Then the media was exchanged with 50 µL/well of drug solution in PBS, and the plates were incubated for 30 min in the dark at 37 °C. Then, 50 µL/well of the calcein-AM solution in PBS was added into each well up to the final concentration of 1 µM, the plates were incubated for another 15 min, and the green fluorescence was directly measured using Infinite F200PRO reader (TECAN) at λ_ex_ = 494 nm and λ_em_ = 517 nm. The viability of the treated cells was controlled by the MTS assay, as previously reported, with slight modifications [39]. In brief, 50 µL of culture media containing the MTS solution was added into each well instead of calcein-AM solution, the plates were incubated for 30 min, and the viability was measured using an Infinite F200PRO reader (TECAN).

### 4.9. Western Blotting

Western blot analysis was performed as described previously [40]. In brief, cells were seeded in the Petri dishes (1 × 10^6^/dish), incubated overnight, followed by 48 h incubation with or without drugs. Cells were harvested by scratching, washing, and lysing using a RIPA lysis buffer containing protease and phosphatase inhibitors. The proteins were subjected to further separation using PAGE and detected using the primary and secondary antibodies listed in Supplementary File S1. The original photos of the Western blotting membranes are represented in Supplementary Figures S1–S6. The quantification of the band intensity has been performed using Image lab software V. 6.1.0. build 7 Standard edition (Bio-Rad Laboratories, Inc., Hercules, CA, USA).

### 4.10. Drug Combinational Studies

The synergistic, additive, or antagonistic effects of the drug combinations were investigated using a Chou–Talalay method [41,42] as described previously [43]. Cell lines were treated with individual drugs or their combinations at a constant molar ratio corresponding to the ratio of IC_50_s (see Table 3). Following 48 h incubation, the cell viability was evaluated using an MTT assay. The generated data were analyzed with CompuSyn v.1.0 (ComboSyn Inc., Paramus, NJ, USA) software, and a combinational index (CI) was calculated. CI > 1.45 was assumed as antagonism; CI = 0.75~1.45 corresponds to additive effect; CI < 0.75 refers to synergism.

### 4.11. DNA Fragmentation and Cell Cycle Analysis

The experiments were performed as previously reported [38]. In brief, 0.2 × 10^6^ cells/well were seeded in 6-well plates in 2 mL/well, incubated overnight, and treated with the tested compounds in fresh culture media (2 mL/well) for 48 h. Then, cells were harvested using trypsination, fixed with 70% EtOH overnight, stained with propidium iodide (PI), and analyzed using the flow cytometry technique. For the analysis, the FACS Calibur (BD Bioscience, San Jose, CA, USA) instrument and BD Bioscience Cell Quest Pro v.5.2.1. software (BD Bioscience) were used.

### 4.12. Global Proteome Screening Analysis

Global proteome screening analysis of PC3 and PC3-DR cells was performed as previously reported [44]. For details, please see Supplementary File S1. In brief, the cells were seeded (2 × 10^6^ cells per T75 culture bottle in 20 mL/bottle) and treated for 48 h with drugs or vehicles. The drug combinations used for treatment are “TIP1” (Cis: Ifo: Pac = 5 µM: 2.5 mM: 125 nM) and “TIP2” (Cis: Ifo: Pac = 10 µM: 5 mM: 250 nM). The cells were harvested by scratching, washing, and lysing. The protein extracts were consequently heat-denaturated, reduced, alkylated, and digested by trypsin. Peptides were chromatographically separated and identified using LC-MS/MS technique in data-dependent acquisition (DDA) mode. For protein identification, the search engine Sequest, integrated into the Proteome Discoverer software version 2.4 (Thermo Fisher Scientific, Bremen, Germany) using the human Uniprot protein database (EMBL, Hinxton Cambridge, U.K., released April 2020), was used, and label-free quantification with a match between runs was performed (Table S1) (For details please see Methods of proteomics/Supplementary information). The proteins having │log_2_(fold change)│ > 1 (i.e., 2 < fold change < 0.5) and *p* ≤ 0.05 (Tables S2–S6) further proceeded to bioinformatical analysis, which was performed using STRING v.11.5 software [45]. The data were subjected to functional enrichment analysis to identify the relevant altered biological processes as well as to the protein network analysis to reveal the possible affected master regulators (Tables S7–S12). Additionally, the detection of overrepresented pathways was performed using clusterProfiler software v4.05 (https://bioconductor.org/packages/release/bioc/html/clusterProfiler.html, accessed on 6 June 2022, Bioconductor project) [46] in combination with the Molecular Signatures database v7.5.1 (http://www.gsea-msigdb.org/gsea/msigdb, accessed on 6 June 2022) [47] and the Gene Ontology (http://geneontology.org/, accessed on 6 June 2022) [48] databases. In this analysis, the Benjamini–Hochberg correction was applied to obtain false discovery rates (FDR). A protein was considered significantly differentially expressed if the corresponding │log_2_(fold change)│ > 1 and the FDR < 0.1.

### 4.13. Statistical Analyses

Patient and treatment characteristics of the clinical cohort were reported as medians and quartiles (continuous variables) or frequencies and proportions (categorical variables). The Kaplan–Meier approach was employed to depict PFS and OS via STATA^®^ statistical software (version 14; StataCorp LP, College Station, TX, USA). Statistical analysis of in vitro data was performed using GraphPad Prism software v. 5.01 (GraphPad Prism software Inc., La Jolla, CA, USA) using two types of analyses: the ANOVA (one-way analysis of variance) test followed by a post hoc Dunnett’s test. Data are presented as mean ± SD (standard deviation). The differences were considered to be statistically significant if *p* < 0.05 (difference between the sample and control: * *p* < 0.05; ** *p* < 0.01; *** *p* < 0.001; difference between two samples: ^#^ *p* < 0.05; ^##^ *p* < 0.01; ^###^ *p* < 0.001).

## 5. Conclusions

In summary, the current study provides a clinical and mechanistic rationale for the combination of Cis, Ifo, and Pac as a treatment regimen that has promising activity in patients with AVPC, mostly with manageable toxicity. In vitro studies have confirmed the efficacy of Cis and Ifo, in particular, when resistance to Doce or neuroendocrine features is present. While the effect of Pac is less clear, Cis and Ifo may be a useful new alternative, especially for patients with suspected AVPC and resistance to Doce. Based on the preclinical findings, we now offer the combination of Cis and Ifo as a salvage therapy to patients with AVPC on individual bases. A clinical trial for the prospective evaluation of this combination is in preparation.

## Figures and Tables

**Table 1:** Baseline characteristics of mCRPC patients treated with TIP.

Patient Baseline Characteristics (n = 17)	
Age, years [median (min; max)]	65 (55;75)
Primary metastatic at diagnosis [n (%)]	6 (37)
Metastatic sites ^1^ [n %] Liver Other visceral sites (lung, adrenal gland, brain) Bone	14 (82) 8 (47) 13 (76)
Prostate-specific antigen (PSA) ^1^, ng/mL [median (IQR ^2^)]	77 (10–640)
Alkaline phosphatase ^1^, µg/L [median (IQR ^2^)]	205 (131–440)
Lactate dehydrogenase (ldh) ^1^, iu/L [median (IQR ^2^)]	903 (285–1728)
Neurone-specific enolase ^1^ (NSE), u/L Median value of all patients [median (IQR ^2^)] Patients with NSE elevation [n %]	33.2 (19–52) 14 (82)
Previous treatment regimens Previous applied treatment lines [median (IQR ^2^)] Docetaxel [%] Cabazitaxel [%] Abiraterone and/or enzalutamide [%] Others [%]	4 (3–6) 100 65 82 41

^1^ At treatment initiation; ^2^ IQR: interquartile range.

**Table 2:** Clinical results of mCRPC patients treated with TIP.

Treatment Summary (n = 17)	
Dosage of chemotherapy (cycle 1) Number of cycles received, median (IQR ^1^) Paclitaxel, mg/sqm, median (IQR ^1^), applied in a single dose Ifosfamide, mg/sqm, split into 2–3 doses [median (IQR ^1^)] Cisplatin ^2^, mg/sqm, split into 2–3 doses [median (IQR ^1^)] Median treatment duration, months, median (IQR ^1^)	3 (2–6) 140 (132.5–162.5) 2400 (2400–3000) 40 (40–60) 3.5 (1.4–3.8)
Treatment discontinuation, n (%) Overall Progressive disease Adverse events (PNP, infection, ifosfamide-induced psychosis) Treatment completion of 6 cycles, n (%)	12 (71) 8 (47) 4 (24) 5 (39)
Radiological assessment (at completion of 6 cycles), n (%) Partial remission Mixed response Stable disease Progressive disease	2 (11.8) 1 (6) 1 (6) 1 (6)
PSA response ^3^ > 30%, n/n PSA elevated at baseline (%)	6/13 (46)
LDH reduction ^3^ > 50%, n/n LDH elevated at baseline (%)	8/16 (50)
NSE reduction ^3^ > 30%, n/n with NSE elevated at baseline (%)	8/13 (62)

^1^ IQR: interquartile range, ^2^ 4 pts received carboplatin AUC 4 due to hearing loss or renal insufficiency; ^3^ at any time during therapy.

**Table 3:** Cytotoxic activity of the drugs in vitro. IC_50_ of cisplatin (Cis), ifosfamide (Ifo), and paclitaxel (Pac) were evaluated in various prostate cancer cell lines following 48 h of treatment using an MTT assay. The data are represented as mean ± standard deviation.

Cell Line	Drug			
	Cis [µM]	Ifo [mM]	Pac [nM]	Doce [nM]
PC3	28.63 ± 4.32	17.45 ± 3.6	290.96 ± 98.37	7.49 ± 7.09
PC3-DR	4.71 ± 1.14	12.38 ± 3.92	552.46 ± 81.58	324.9 ± 21.7
DU145	15.33 ± 7.74	12.18 ± 0.812	14.93 ± 1.32	13.29 ± 1.04
DU145-DR	2.29 ± 1.12	8.76 ± 2.49	119.2 ± 22.2	372.5 ± 53.9
LASCPC-01	0.94 ± 0.06	3.13 ± 0.61	7.16 ± 1.49	-
22Rv1	4.7 ± 1.06	4.83 ± 1.82	11.51 ± 2.47	-
LNCaP	14.55 ± 6.78	4.99 ± 0.82	6.02 ± 0.24	-

## Data Availability

All the data are available from the corresponding author on request.

## Supplementary Materials

The following supporting information can be downloaded at: https://www.mdpi.com/article/10.3390/ijms232314948/s1.

## References

[1] Halabi S., Kelly W.K., Ma H., Zhou H., Solomon N.C., Fizazi K., Tangen C.M., Rosenthal M., Petrylak D.P., Hussain M. (2016). Meta-Analysis Evaluating the Impact of Site of Metastasis on Overall Survival in Men With Castration-Resistant Prostate Cancer. J. Clin. Oncol..

[2] Aparicio A.M., Harzstark A.L., Corn P.G., Wen S., Araujo J.C., Tu S.M., Pagliaro L.C., Kim J., Millikan R.E., Ryan C. (2013). Platinum-based chemotherapy for variant castrate-resistant prostate cancer. Clin. Cancer Res..

[3] Motzer R.J., Sheinfeld J., Mazumdar M., Bains M., Mariani T., Bacik J., Bajorin D., Bosl G.J. (2000). Paclitaxel, ifosfamide, and cisplatin second-line therapy for patients with relapsed testicular germ cell cancer. J. Clin. Oncol..

[4] Albers P., Albrecht W., Algaba F., Bokemeyer C., Cohn-Cedermark G., Fizazi K., Horwich A., Laguna M.P. (2021). Tandstad EAU Guidelines on Testicular Cancer: Update 2021.

[5] Schmid S., Omlin A., Higano C., Sweeney C., Martinez Chanza N., Mehra N., Kuppen M.C.P., Beltran H., Conteduca V., Vargas Pivato de Almeida D. (2020). Activity of Platinum-Based Chemotherapy in Patients With Advanced Prostate Cancer With and Without DNA Repair Gene Aberrations. JAMA Netw. Open.

[6] Sternberg C.N., Petrylak D.P., Sartor O., Witjes J.A., Demkow T., Ferrero J.M., Eymard J.C., Falcon S., Calabro F., James N. (2009). Multinational, double-blind, phase III study of prednisone and either satraplatin or placebo in patients with castrate-refractory prostate cancer progressing after prior chemotherapy: The SPARC trial. J. Clin. Oncol..

[7] Teply B.A., Antonarakis E.S. (2017). Treatment strategies for DNA repair-deficient prostate cancer. Expert Rev. Clin. Pharmacol..

[8] Corn P.G., Heath E.I., Zurita A., Ramesh N., Xiao L., Sei E., Li-Ning-Tapia E., Tu S.M., Subudhi S.K., Wang J. (2019). Cabazitaxel plus carboplatin for the treatment of men with metastatic castration-resistant prostate cancers: A randomised, open-label, phase 1-2 trial. Lancet Oncol..

[9] Kentepozidis N., Soultati A., Giassas S., Vardakis N., Kalykaki A., Kotsakis A., Papadimitraki E., Pantazopoulos N., Bozionellou V., Georgoulias V. (2012). Paclitaxel in combination with carboplatin as salvage treatment in patients with castration-resistant prostate cancer: A Hellenic oncology research group multicenter phase II study. Cancer Chemother. Pharmacol..

[10] Urakami S., Igawa M., Kikuno N., Yoshino T., Kishi H., Shigeno K., Shiina H. (2002). Combination chemotherapy with paclitaxel, estramustine and carboplatin for hormone refractory prostate cancer. J. Urol..

[11] Berry W.R., Hathorn J.W., Dakhil S.R., Loesch D.M., Jackson D.V., Gregurich M.A., Newcomb-Fernandez J.K., Asmar L. (2004). Phase II randomized trial of weekly paclitaxel with or without estramustine phosphate in progressive, metastatic, hormone-refractory prostate cancer. Clin. Prostate Cancer.

[12] Fujiwara M., Akamatsu S., Sumiyoshi T., Segawa T., Mizuno K., Yoshino T., Goto T., Sawada A., Saito R., Kobayashi T. (2019). Efficacy and Safety of Carboplatin Plus Paclitaxel as the First-, Second-, and Third-line Chemotherapy in Men with Castration-resistant Prostate Cancer. Clin. Genitourin. Cancer.

[13] Jeske S., Tagawa S.T., Olowokure O., Selzer J., Giannakakou P., Nanus D.M. (2011). Carboplatin plus paclitaxel therapy after docetaxel in men with metastatic castrate resistant prostate cancer. Urol. Oncol..

[14] Ifosfamide (1994). BC Cancer Agency Cancer Drug Manual.

[15] Hervonen P., Tulijoki T., Kellokumpu-Lehtinen P. (2012). No additional benefit of adding ifosfamide to docetaxel in castration-resistant metastatic prostate cancer. Anticancer Res..

[16] Fujita K., Matsushima H., Nakano M., Kaneko T. (1994). Ifosfamide in combined hormonochemotherapy on prostate cancer. Gan Kagaku Ryoho Cancer Chemother..

[17] Nait Slimane S., Marcel V., Fenouil T., Catez F., Saurin J.C., Bouvet P., Diaz J.J., Mertani H.C. (2020). Ribosome Biogenesis Alterations in Colorectal Cancer. Cells.

[18] Xiao J.F., Zhou B., Ressom H.W. (2012). Metabolite identification and quantitation in LC-MS/MS-based metabolomics. Trends Analyt. Chem..

[19] Duran G.E., Derdau V., Weitz D., Philippe N., Blankenstein J., Atzrodt J., Semiond D., Gianolio D.A., Mace S., Sikic B.I. (2018). Cabazitaxel is more active than first-generation taxanes in ABCB1(+) cell lines due to its reduced affinity for P-glycoprotein. Cancer Chemother Pharmacol..

[20] Huang P.H., Cook R., Mittnacht S. (2015). RB in DNA repair. Oncotarget.

[21] Smith J., Mun Tho L., Xu N., Gillespie D.A., Vande Woude G.F., Klein G. (2010). Chapter 3—The ATM–Chk2 and ATR–Chk1 Pathways in DNA Damage Signaling and Cancer. Advances in Cancer Research.

[22] Tsaur I., Heidegger I., Kretschmer A., Borgmann H., Gandaglia G., Briganti A., de Visschere P., Mathieu R., Valerio M., van den Bergh R. (2019). Aggressive variants of prostate cancer—Are we ready to apply specific treatment right now?. Cancer Treat. Rev..

[23] Merkens L., Sailer V., Lessel D., Janzen E., Greimeier S., Kirfel J., Perner S., Pantel K., Werner S., von Amsberg G. (2022). Aggressive variants of prostate cancer: Underlying mechanisms of neuroendocrine transdifferentiation. J. Exp. Clin. Cancer Res..

[24] Locke V.L., Davey R.A., Davey M.W. (2003). Modulation of drug and radiation resistance in small cell lung cancer cells by paclitaxel. Anticancer Drugs.

[25] Ruiz de Porras V., Wang X.C., Palomero L., Marin-Aguilera M., Sole-Blanch C., Indacochea A., Jimenez N., Bystrup S., Bakht M., Conteduca V. (2021). Taxane-induced Attenuation of the CXCR2/BCL-2 Axis Sensitizes Prostate Cancer to Platinum-based Treatment. Eur. Urol..

[26] Parker R.J., Dabholkar M.D., Lee K.B., Bostick-Bruton F., Reed E. (1993). Taxol effect on cisplatin sensitivity and cisplatin cellular accumulation in human ovarian cancer cells. J. Natl. Cancer Inst. Monogr..

[27] Waldman T., Zhang Y., Dillehay L., Yu J., Kinzler K., Vogelstein B., Williams J. (1997). Cell-cycle arrest versus cell death in cancer therapy. Nat. Med..

[28] Uysal-Onganer P., Kawano Y., Caro M., Walker M.M., Diez S., Darrington R.S., Waxman J., Kypta R.M. (2010). Wnt-11 promotes neuroendocrine-like differentiation, survival and migration of prostate cancer cells. Mol. Cancer.

[29] de Bono J., Mateo J., Fizazi K., Saad F., Shore N., Sandhu S., Chi K.N., Sartor O., Agarwal N., Olmos D. (2020). Olaparib for Metastatic Castration-Resistant Prostate Cancer. N. Engl. J. Med..

[30] Heck M.M., Retz M., D’Alessandria C., Rauscher I., Scheidhauer K., Maurer T., Storz E., Janssen F., Schottelius M., Wester H.J. (2016). Systemic Radioligand Therapy with (177)Lu Labeled Prostate Specific Membrane Antigen Ligand for Imaging and Therapy in Patients with Metastatic Castration Resistant Prostate Cancer. J. Urol..

[31] Sartor O., de Bono J., Chi K.N., Fizazi K., Herrmann K., Rahbar K., Tagawa S.T., Nordquist L.T., Vaishampayan N., El-Haddad G. (2021). Lutetium-177-PSMA-617 for Metastatic Castration-Resistant Prostate Cancer. N. Engl. J. Med..

[32] von Amsberg G., Alsdorf W., Karagiannis P., Coym A., Kaune M., Werner S., Graefen M., Bokemeyer C., Merkens L., Dyshlovoy S.A. (2022). Immunotherapy in Advanced Prostate Cancer-Light at the End of the Tunnel?. Int. J. Mol. Sci..

[33] Agarwal N., McGregor B., Maughan B.L., Dorff T.B., Kelly W., Fang B., McKay R.R., Singh P., Pagliaro L., Dreicer R. (2022). Cabozantinib in combination with atezolizumab in patients with metastatic castration-resistant prostate cancer: Results from an expansion cohort of a multicentre, open-label, phase 1b trial (COSMIC-021). Lancet Oncol..

[34] Sweeney C., Bracarda S., Sternberg C.N., Chi K.N., Olmos D., Sandhu S., Massard C., Matsubara N., Alekseev B., Parnis F. (2021). Ipatasertib plus abiraterone and prednisolone in metastatic castration-resistant prostate cancer (IPATential150): A multicentre, randomised, double-blind, phase 3 trial. Lancet.

[35] Shorning B.Y., Dass M.S., Smalley M.J., Pearson H.B. (2020). The PI3K-AKT-mTOR Pathway and Prostate Cancer: At the Crossroads of AR, MAPK, and WNT Signaling. Int. J. Mol. Sci..

[36] Beltran H., Oromendia C., Danila D.C., Montgomery B., Hoimes C., Szmulewitz R.Z., Vaishampayan U., Armstrong A.J., Stein M., Pinski J. (2019). A Phase II Trial of the Aurora Kinase A Inhibitor Alisertib for Patients with Castration-resistant and Neuroendocrine Prostate Cancer: Efficacy and Biomarkers. Clin. Cancer Res..

[37] Puhr M., Hoefer J., Schäfer G., Erb H.H.H., Oh S.J., Klocker H., Heidegger I., Neuwirt H., Culig Z. (2012). Epithelial-to-mesenchymal transition leads to docetaxel resistance in prostate cancer and is mediated by reduced expression of miR-200c and miR-205. Am. J. Pathol..

[38] Dyshlovoy S.A., Tabakmakher K.M., Hauschild J., Shchekaleva R.K., Otte K., Guzii A.G., Makarieva T.N., Kudryashova E.K., Fedorov S.N., Shubina L.K. (2016). Guanidine alkaloids from the marine sponge *Monanchora pulchra* show cytotoxic properties and prevent EGF-induced neoplastic transformation in vitro. Mar. Drugs.

[39] Dyshlovoy S.A., Rast S., Hauschild J., Otte K., Alsdorf W.H., Madanchi R., Kalinin V.I., Silchenko A.S., Avilov S.A., Dierlamm J. (2017). Frondoside A induces AIF-associated caspase-independent apoptosis in Burkitt lymphoma cells. Leuk. Lymphoma.

[40] Dyshlovoy S.A., Pelageev D.N., Hauschild J., Sabutskii Y.E., Khmelevskaya E.A., Krisp C., Kaune M., Venz S., Borisova K.L., Busenbender T. (2020). Inspired by sea urchins: Warburg effect mediated selectivity of novel synthetic non-glycoside 1,4-naphthoquinone-6S-glucose conjugates in prostate cancer. Mar. Drugs.

[41] Chou T.-C. (2006). Theoretical basis, experimental design, and computerized simulation of synergism and antagonism in drug combination studies. Pharmacol. Rev..

[42] Chou T.-C. (2010). Drug combination studies and their synergy quantification using the Chou-Talalay method. Cancer Res..

[43] Dyshlovoy S.A., Madanchi R., Hauschild J., Otte K., Alsdorf W.H., Schumacher U., Kalinin V.I., Silchenko A.S., Avilov S.A., Honecker F. (2017). The marine triterpene glycoside frondoside A induces p53-independent apoptosis and inhibits autophagy in urothelial carcinoma cells. BMC Cancer.

[44] Dyshlovoy S.A., Pelageev D.N., Hauschild J., Borisova K.L., Kaune M., Krisp C., Venz S., Sabutskii Y.E., Khmelevskaya E.A., Busenbender T. (2019). Successful Targeting of the Warburg Effect in Prostate Cancer by Glucose-Conjugated 1,4-Naphthoquinones. Cancers.

[45] Szklarczyk D., Gable A.L., Nastou K.C., Lyon D., Kirsch R., Pyysalo S., Doncheva N.T., Legeay M., Fang T., Bork P. (2020). The STRING database in 2021: Customizable protein-protein networks, and functional characterization of user-uploaded gene/measurement sets. Nucleic Acids Res..

[46] Yu G., Wang L.-G., Han Y., He Q.-Y. (2012). clusterProfiler: An R Package for Comparing Biological Themes Among Gene Clusters. OMICS A J. Integr. Biol..

[47] Liberzon A., Birger C., Thorvaldsdóttir H., Ghandi M., Mesirov J.P., Tamayo P. (2015). The Molecular Signatures Database (MSigDB) hallmark gene set collection. Cell Syst..

[48] Ashburner M., Ball C.A., Blake J.A., Botstein D., Butler H., Cherry J.M., Davis A.P., Dolinski K., Dwight S.S., Eppig J.T. (2000). Gene ontology: Tool for the unification of biology. The Gene Ontology Consortium. Nat. Genet..

