# Supplementary material for: Salvage Chemotherapy with Cisplatin, Ifosfamide, and Paclitaxel in Aggressive Variant of Metastatic Castration-Resistant Prostate Cancer
Source: Int J Mol Sci. 2022 Nov 29;23(23):14948. doi: 10.3390/ijms232314948 (PMC9738104; doi:10.3390/ijms232314948)
Supplement: Supplementary file 1 [file ijms-23-14948-s001.zip › captions of Figures and Tables for the supplementary.pdf]

Supplementary File 1: List of primary and secondary antibodies used; Methods of proteomics; **Figure S1**. Original photos of the Western blotting membranes represented in Figure 5A; **Figure S2**. Original photos of the Western blotting membranes represented in Figure 6B; **Figure S3**. Original photos of the Western blotting membranes represented in Figure 7A; **Figure S4**. Original photos of the Western blotting membranes represented in Figure 7A (continuation); **Figure S5**. Original photos of the Western blotting membranes represented in Figure 7B; **Figure S6**. Original photos of the Western blotting membranes represented in Figure 7B (continuation); **Figure S7**. Effect of Cis and TIP on the protein expression in PC3-DR and 22Rv1 cells; **Figure S8**. Expression of some proteins in PC3 and PC3-DR cells; **Figure S9**. Effect of TIP on the protein expression in PC3-DR cells; **Figure S10**. Evaluation of DNA double-strand breaks (DSB) in the cells following the treatment; **Table S14**. Number of colocalized γH2AX/53BP1 foci considered a marker of DSB.

Supplementary File 2: Table S1. Full list of the identified proteins; **Table S2.** Proteins significantly and differently expressed in PC3-DR cells versus PC3 cells; **Table S3.** Proteins significantly and differently expressed in PC3 cells treated with TIP1 versus control; **Table S4.** Proteins significantly and differently expressed in PC3 cells treated with TIP2 versus control; **Table S5.** Proteins significantly and differently expressed in PC3-DR cells treated with TIP1 versus control; **Table S6.** Proteins significantly and differently expressed in PC3-DR cells treated with TIP2 versus control.

Supplementary File 3: Table S7. Protein rank-based analysis. Functional enrichments, untreated PC3-DR cells versus PC3 cells; **Table S8**. Functional enrichments, untreated PC3-DR cells versus PC3 cells; **Table S9.** Functional enrichments, PC3 cells treated with TIP1 versus control; **Table S10.** Functional enrichments, PC3 cells treated with TIP2 versus control; **Table S11.** Functional enrichments, PC3-DR cells treated with TIP1 versus control; **Table S12.** Functional enrichments, PC3-DR cells treated with TIP2 versus control; **Table S13.** Analysis of the overrepresented pathways, PC3-DR cells treated with TIP2 versus PC3 cells treated with TIP2.
