# Supplementary material for: Salvage Chemotherapy with Cisplatin, Ifosfamide, and Paclitaxel in Aggressive Variant of Metastatic Castration-Resistant Prostate Cancer
Source: Int J Mol Sci. 2022 Nov 29;23(23):14948. doi: 10.3390/ijms232314948 (PMC9738104; doi:10.3390/ijms232314948)
Supplement: Supplementary file 1 [file ijms-23-14948-s001.zip › Supplementary file S1.pdf]

List of primary and secondary antibodies used

Antibodies	Clonality	Source	Cat.-No.	Dilution	Manufacturer
Rb	mAb	mouse	9309	1:1000	Cell Signaling
phospho-Rb (Ser807/811)	mAb	rabbit	8516	1:1000	Cell Signaling
anti-cleaved Caspase-3	mAb	rabbit	9664	1:1000	Cell Signaling
Sox2 (D6D9)	mAb	rabbit	3579	1:1000	Cell Signaling
anti-p21$^{Waf1/Cip1}$	mAb	rabbit	2947	1:1000	Cell Signaling
anti-PARP	pAb	rabbit	9542	1:1000	Cell Signaling
E-cadherin	mAb	mouse	14472	1:1000	Cell Signaling
N-cadherin	mAb	rabbit	13116	1:1000	Cell Signaling
Enolase-2	mAb	rabbit	8171	1:1000	Cell Signaling
Synaptophysin	mAb	rabbit	36406	1:1000	Cell Signaling
Oct-4	pAb	rabbit	2750	1:1000	Cell Signaling
Chromogranin A	mAb	rabbit	85798	1:1000	Cell Signaling
anti-α-Tubulin	mAb	mouse	T5168	1:5000	Sigma-Aldrich
anti-β-Actin-HRP	pAb	goat	sc-1616	1:10000	Santa Cruz
anti-Survivin	pAb	rabbit	NB500-201	1:1000	Novus Biologicals
ATR	mAb	mouse	sc-515173	1:1000	Santa Cruz
phospho-ATR (Thr1989)	pAb	rabbit	58014	1:1000	Cell Signaling
ATM (2C1)	mAb	mouse	GTX70103	1:1000	GeneTEX
phospho-ATM (pS1981)	pAb	rabbit	GTX132146	1:1000	GeneTEX
Mre11 (12D7)	mAb	mouse	ab214	1:1000	abcam
CHK1 (2G1D5)	mAb	mouse	2360	1:1000	Cell Signaling
phospho-CHK1 (pS345) (133D3)	mAb	rabbit	2348	1:1000	Cell Signaling
CHK2	mAb	mouse	611570	1:1000	BD
phospho-CHK2 (Thr68)	pAb	rabbit	2661	1:1000	Cell Signaling
Ku 70	mAb	mouse	NB100-1915	1:1000	Novus Biologicals
KU80	pAb	rabbit	2753	1:1000	Cell Signaling
MDR1/ABCB1 (p-gp)	mAb	mouse	12683	1:1000	Cell Signaling
phospho-S139-H2AX	mAb	Mouse	05-636	1:500	Millipore
53BP1	pAb	rabbit	NB100-304	1:500	Novus Biologicals
anti-rabbit Alexa-fluor488		goat	A32731	1:600	Invitrogen
anti-mouse Alexa-fluor594		donkey	A32744	1:500	Invitrogen
anti-mouse IgG-HRP		sheep	NXA931	1:10000	GE Healthcare
anti-rabbit IgG-HRP		goat	7074	1:5000	Cell Signaling

Methods of proteomics

LC-MS sample preparation

Cell lines were lysed in 100 mM triethylammonium bicarbonate and 1% w/v sodium deoxycholate buffer followed by probe sonication to destroy polynucleotides and heat induce protein denaturing at 99°C for 5 min. Protein concentrations were estimated with a bicinchonic acid (BCA) protein assay (Thermo Fisher Scientific, Bremen, Germany). 20 µg of protein were reduced in presence of 10 mM dithiotreithol (Sigma Aldrich) for 30 min at 60°C followed by cysteine alkylation with 20 mM iodo acetamide (Sigma Aldrich) for 30 min at 37°C in the dark and enzymatic degradation with sequencing grade trypsin (Promega) over night at 37°C. Digestion was quenched with 1% formic acid (FA), precipitated sodium deoxycholate removed by centrifugation for 5 min at 14000 g and the supernatant was dried in a vacuum centrifuge.

LC-MS/MS in Data Dependent and Data Independent mode

Samples were resuspended in 0.1% FA and transferred into a full recovery autosampler vial (Waters). Chromatographic separation was achieved on a nano-UPLC system (Acquity, Waters Corporation, Milford, MS, USA) with a two-buffer-system (buffer A: 0.1% FA in water, buffer B: 0.1% FA in Acetonitrile (ACN), both at pH = 3). Attached to the UPLC was a reversed-phase peptide trapping column (Symmetry C18, 180 µm × 20 mm, 100 Å pore size, 5 µm particle size) for desalting, followed by a reversed-phase capillary separation of the tryptic peptides (BEH C18; 75 µm × 200 mm, 130 Å pore size, 1.7 µm particle size), using a 60 min gradient with increasing ACN concentration from 2% – 30% ACN. The eluting peptides were analyzed on a quadrupole-orbitrap mass spectrometer (QExactive, Thermo Fisher Scientific, Bremen, Germany) in data dependent acquisition (DDA) and data independent acquisition (DIA) for quantification.

In DDA mode, one replicate of each cell line was randomly chosen and 1 µg per sample was used per LC-MS/MS run to build a reference spectral library used for data extraction of samples acquired in DIA mode. For DDA, the 12 most intense ions per precursor scan (1×10^6 ions, 70000 Resolution, 120 ms fill time) were analyzed by MS/MS (HCD at 25 normalized collision energy, $1x10^5$ ions, 17500 Resolution, 50 ms fill time) in a range of 400 – 1300 m/z. A dynamic precursor exclusion of 20 s was used. For DIA, 1 µg of each sample was analyzed using a 30 sequential 20 Da fixed window method covering the mass range from 400 – 1000 m/z. Per cycle, 2 precursor scans ($1x10^6$ ions, 35000 Resolution, 110 ms fill time, m/z range 390 – 1010 m/z) and 30 MS/MS scans (HCD at 28 normalized collision energy, 1×10^6 ions, 17500 Resolution, 50 ms fill time) were performed. After the first precursor scan, 15 MS/MS scans were performed covering the precursor mass range from 400 – 700 m/z followed by the second precursor scan and another 15 MS/MS scans ranging from 700 – 1000 m/z.

LC-MS/MS data processing and analysis

Acquired DDA LC-MS/MS data were searched against the human SwissProt protein data base downloaded from Uniprot protein database (release December 2018, EMBL, Hinxton, Great Britain) using the search engine Sequest integrated in the protein identification software “Proteome Discoverer” (version 2.0, Thermo Fisher Scientific, Bremen, Germany). Mass tolerances for precursors was set to 10 ppm and 0.02 Da for fragments. Carbamidomethylation was set as a fixed modification for cysteine residues and the oxidation of methionine, pyro-glutamate formation at glutamine residues at the peptide N-terminus as well as acetylation of the protein N-terminus, methionine loss at the protein N-terminus and the Acetylation after methionine loss at the protein N-terminus were allowed as variable modifications. Only peptide with a high confidence (false discovery rate < 1% using a decoy data base approach) were accepted as identified.

Proteome Discoverer search results were imported into Skyline quantification software for processing of DIA LC-MS data (version 4.2, MacCoss Lab Software, University of Washington, USA) allowing only high confidence peptides with more than 4 fragment ions. A maximum of 5 fragment ions per peptide were used for information extraction from DIA files for peptides with a dot product of > 0.85. Peptide peak areas were summed to generate protein areas which were then used for relative abundance comparison. Protein areas were imported into a Perseus statistical analysis software (version 1.5.8; Max Planck Institute of Biochemistry; Munich, Germany) (Tyanova et al. Nature Methods. 2016. 13(9):731-40).

Figure S1. Original photos of the Western blotting membranes represented at Figure 5A.

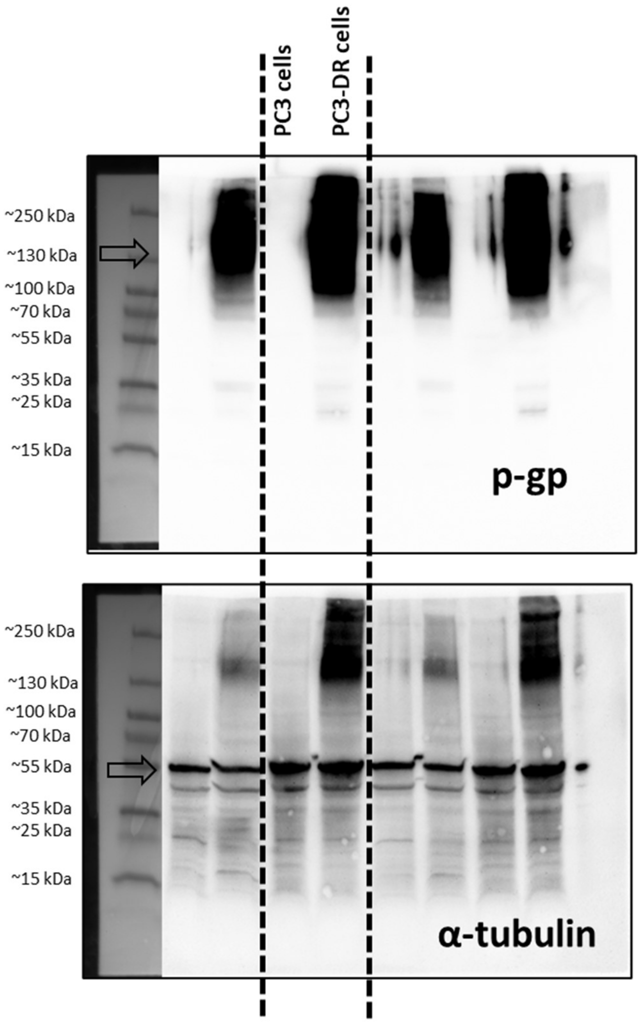

Figure S2. Original photos of the Western blotting membranes represented at Figure 6B.

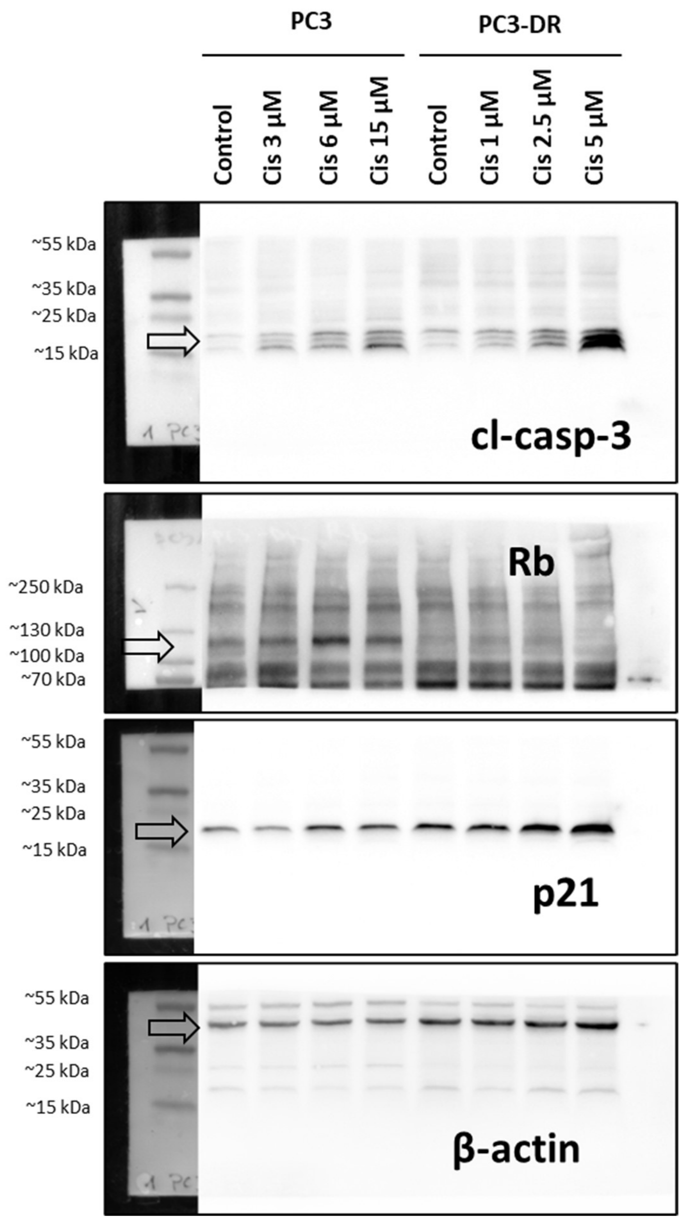

Figure S3. Original photos of the Western blotting membranes represented at Figure 7A.

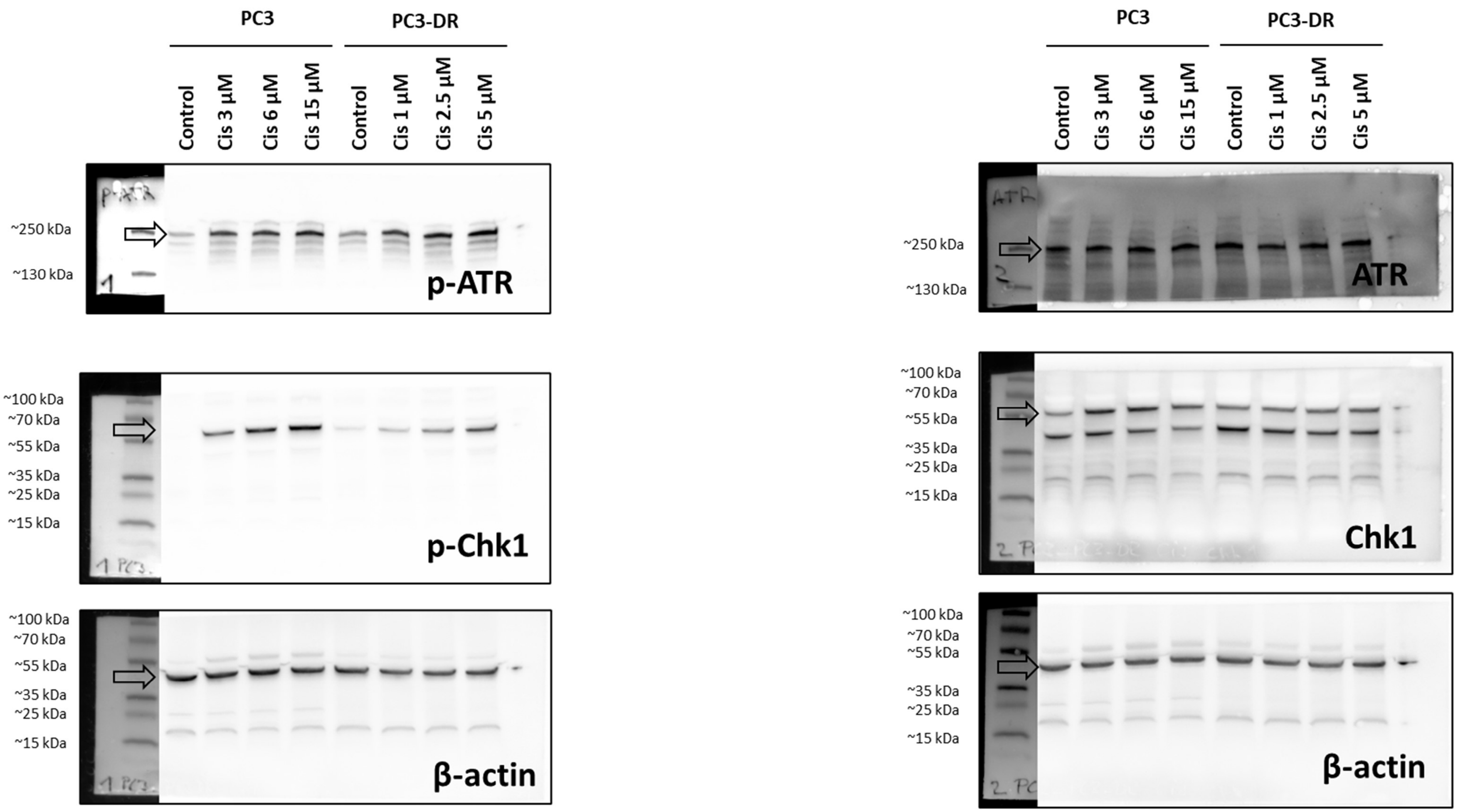

Figure S4. Original photos of the Western blotting membranes represented at Figure 7A (continuation).

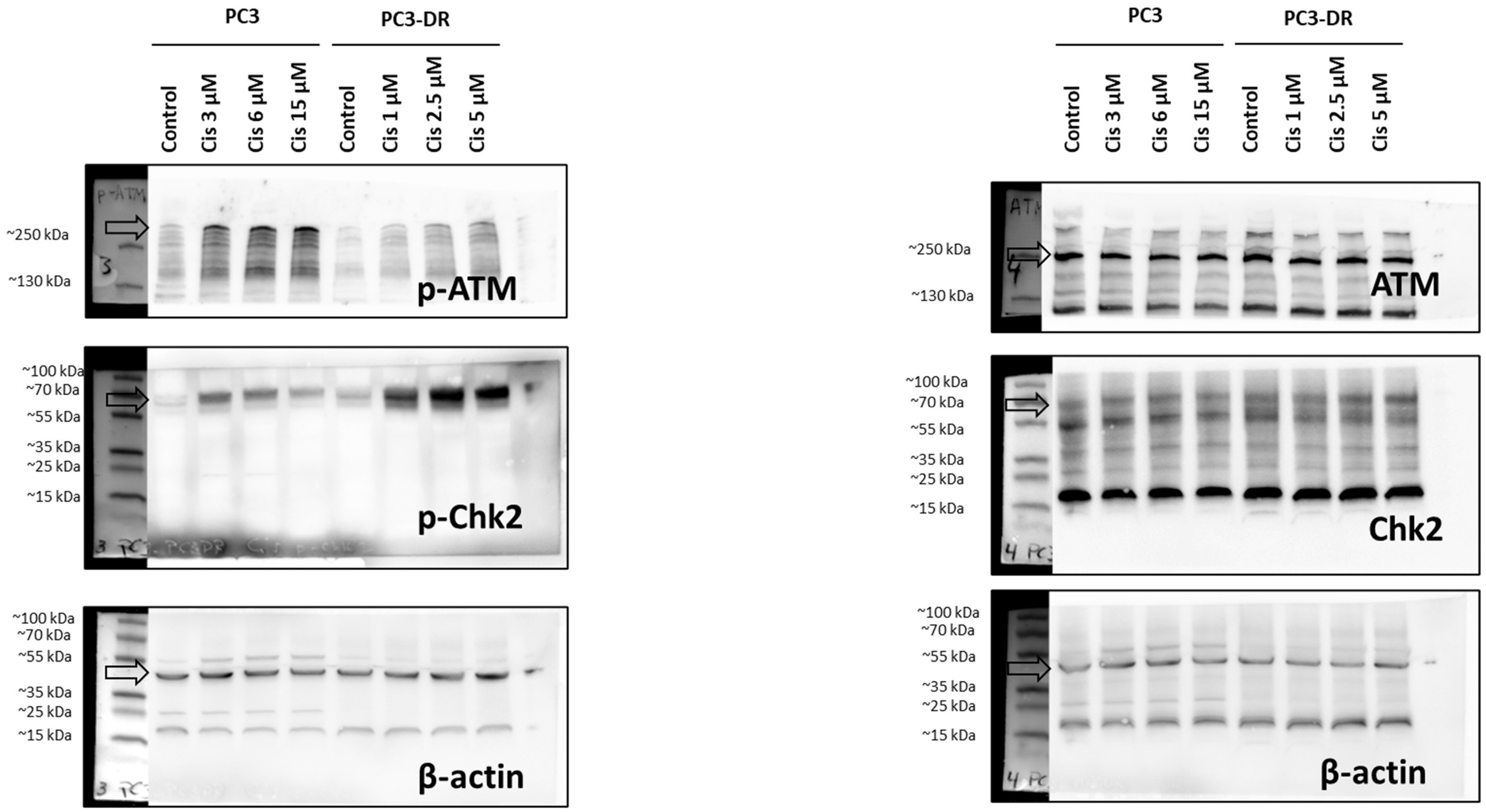

Figure S5. Original photos of the Western blotting membranes represented at Figure 7B.

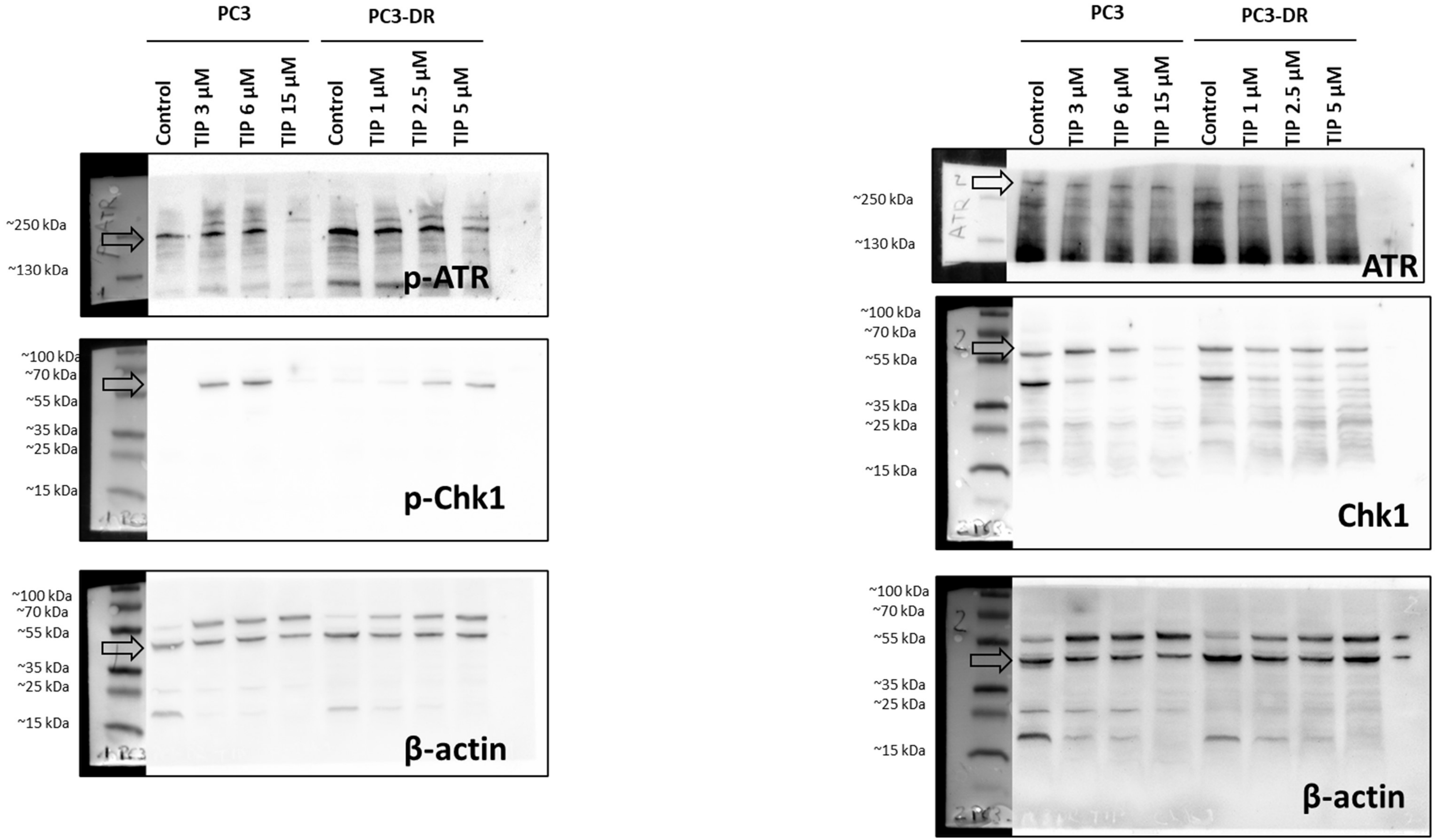

Figure S6. Original photos of the Western blotting membranes represented at Figure 7B (continuation).

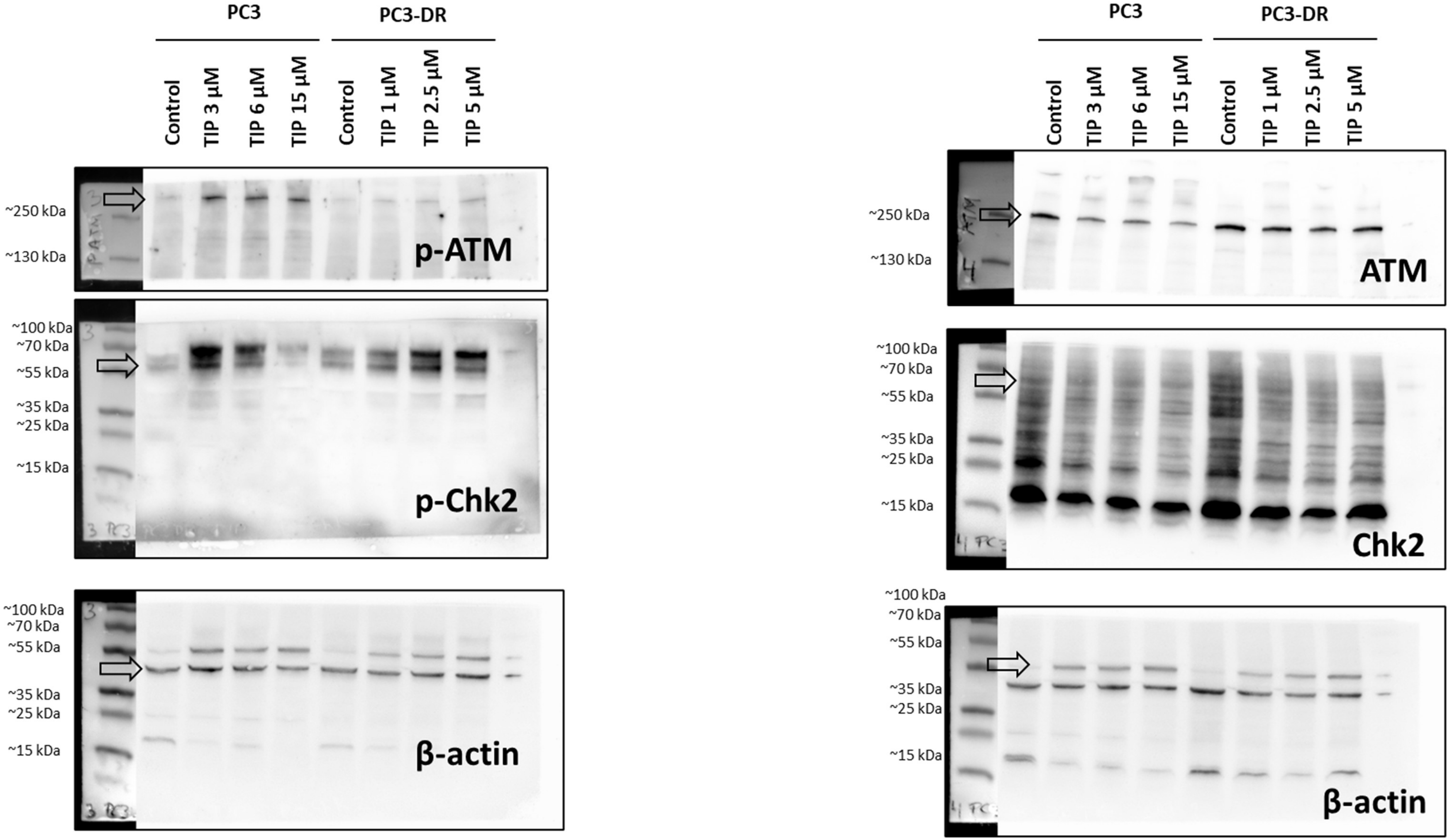

Figure S7. Effect of Cis and TIP on the protein expression in PC3-DR and 22Rv1 cells. The levels of protein expression were analyzed by Western blotting. The cells were treated with indicated concentrations of Cis, Ifo, Pac or their combination (TIP) for 48 h.

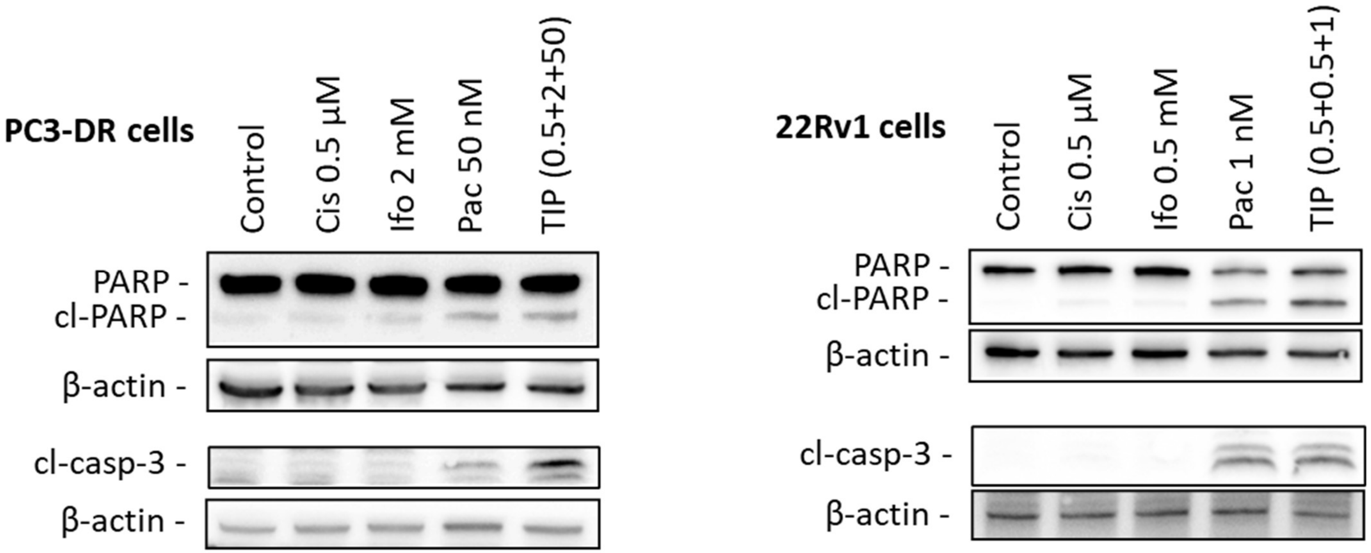

Figure S8. Expression of some proteins in PC3 and PC3-DR cells. The levels of protein expression were analyzed by Western blotting.

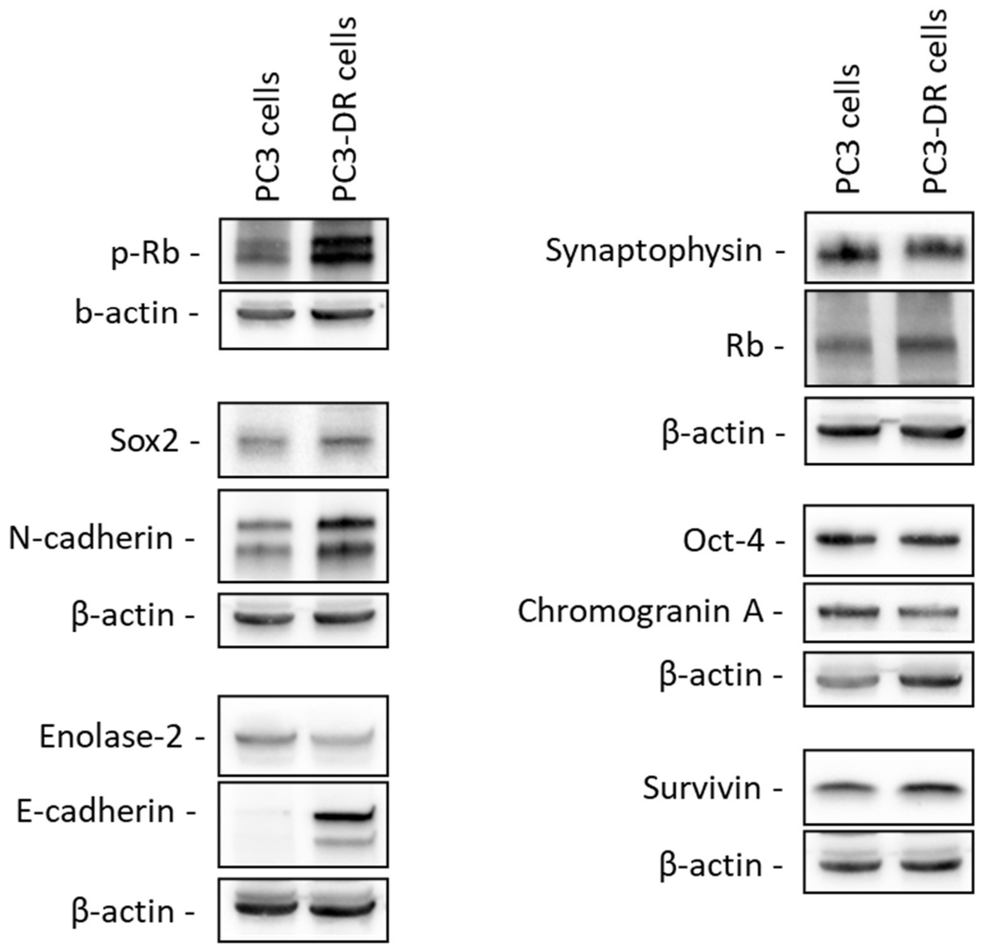

Figure S9. Effect of TIP on the protein expression in PC3-DR cells. The levels of protein expression were analyzed by Western blotting following 48 h of treatment. The cells were treated with indicated concentrations of Cis, Ifo, Pac or their combination (TIP) for 48 h. The following drug concentrations were used: TIP 0.5 (Cis 0.5 µM, Ifo 2 mM, Pac 50 nM), TIP 1 (Cis 1 µM, Ifo 4 mM, Pac 100 nM), TIP 2.5 (Cis 2.5 µM, Ifo 10 mM, Pac 250 nM).

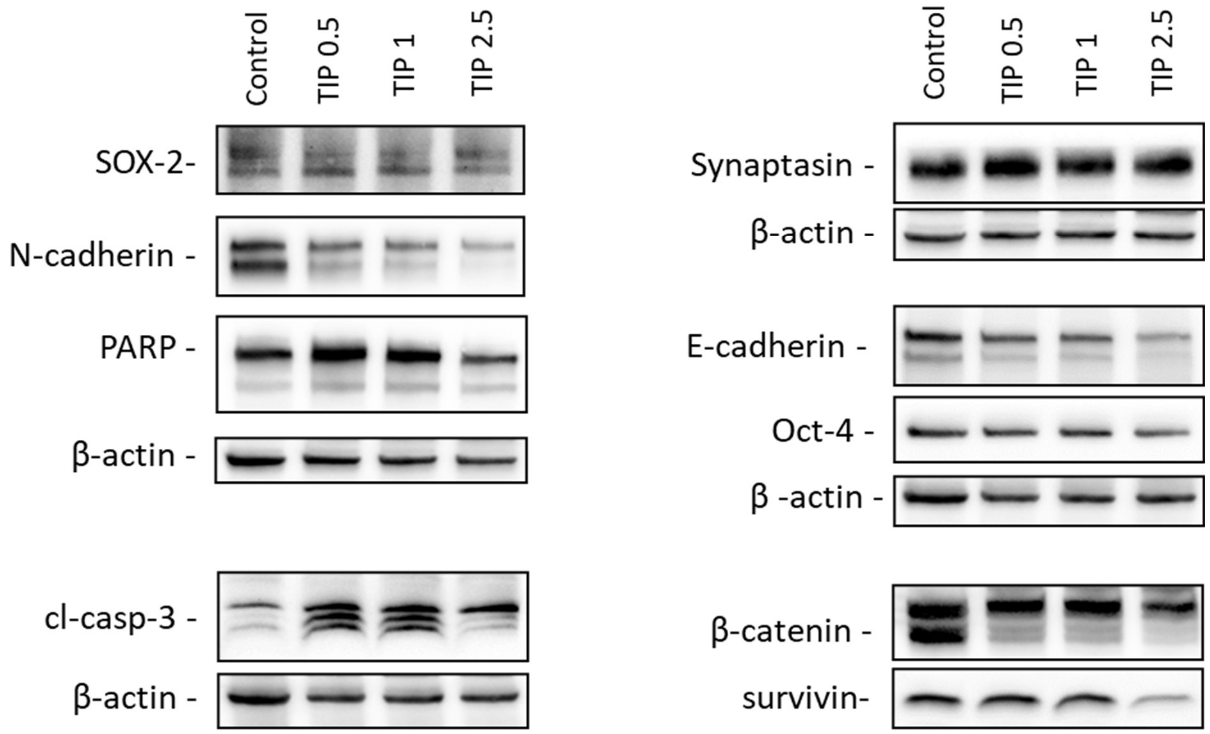

Figure S10. Evaluation of DNA double-strand breaks (DSB) in the cells following the treatment. PC3 and PC3-DR cells were treated with the individual drugs and their combinations for 6 h. 6 h, 24 h and 48 h following the treatment an immunofluorescence double staining for γH2AX (red graphs, upper panel) and 53BP1 (green graphs, upper panel) was performed. Colocalized γH2AX/53BP1 foci were considered as a marker of DSB and were quantified microscopically (orange graphs, upper panel). For each experimental condition at least 100 cells were analyzed. Data are indicated as means ± SD from at least three independent experiments. The original data are presented in the Table S14.

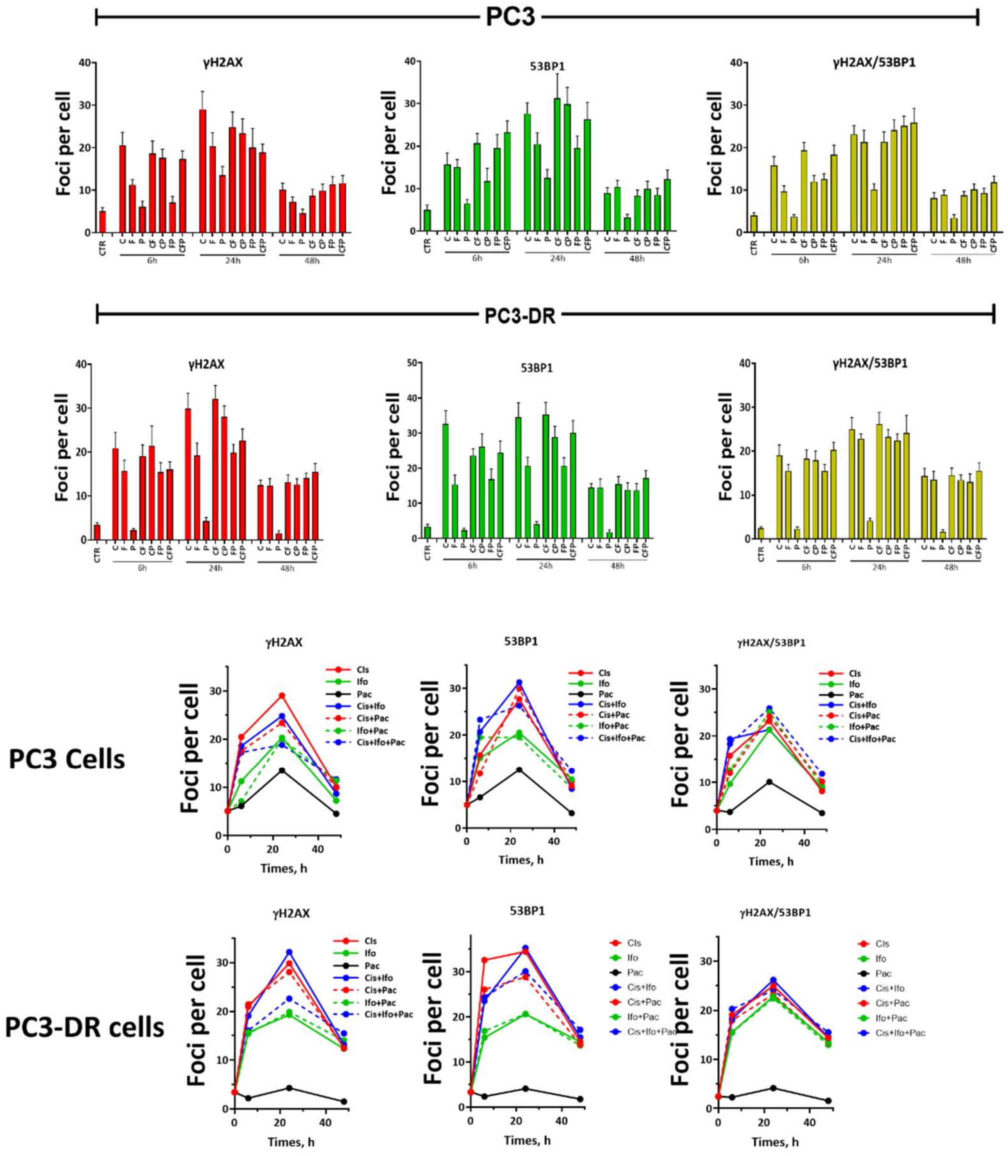

Table S14. Number of co-localized γH2AX/53BP1 foci considered as a marker of DSB. The foci were quantified microscopically. For each experimental condition at least 100 cells were analyzed. Data are indicated as means ± SD (standard deviation) as well as n (number of replicates). Data are presented in as a graphs on Figures 8B and S3.

PC3 cells

	Cis			Ifo			Pac			Cis+Ifo			Cis+Pac			Ifo+Pac			Cis+Ifo+Pac		
	mean	SD	n	mean	SD	n	mean	SD	n	mean	SD	n	mean	SD	n	mean	SD	n	mean	SD	n
0 h	4	0.7395	29	4	0.7395	29	4	0.7395	29	4	0.7395	29	4	0.7395	29	4	0.7395	29	4	0.7395	29
6 h	15.78	2.115	32	9.659	1.346	41	3.694	0.5343	36	19.33	1.838	39	12	1.421	34	12.63	1.211	40	18.31	2.239	42
24 h	23.14	2.089	36	21.3	2.801	33	10.12	1.383	41	21.35	2.381	40	24.13	2.394	39	25.09	2.343	34	25.88	3.397	33
48 h	8.138	1.278	29	8.867	1.119	30	3.462	0.779	26	8.724	0.9724	29	10.17	1.328	24	9.269	1.196	26	11.84	1.468	19

PC3-DR cells

	Cis			Ifo			Pac			Cis+Ifo			Cis+Pac			Ifo+Pac			Cis+Ifo+Pac		
	mean	SD	n	mean	SD	n	mean	SD	n	mean	SD	n	mean	SD	n	mean	SD	n	mean	SD	n
0 h	2.459	0.3668	37	2.459	0.3668	37	2.459	0.3668	37	2.459	0.3668	37	2.459	0.3668	37	2.459	0.3668	37	2.459	0.3668	37
6 h	19.05	2.359	39	15.52	1.442	21	2.286	0.4091	21	18.26	2.05	27	17.96	2.063	24	15.47	1.528	19	20.26	1.752	23
24 h	24.97	2.699	32	22.81	1.119	16	4.16	0.5677	25	26.16	2.65	19	23.21	1.74	19	22.42	1.541	19	24.08	4.07	26
48 h	14.35	1.728	23	13.5	1.916	24	1.579	0.5091	19	14.52	1.605	23	13.4	1.2	20	12.95	1.871	20	15.5	1.812	22
